# Elicitor-driven enhancement of phenolic compounds in geranium callus cultures: phytochemical profiling via LC-MS/MS and biological activities

**DOI:** 10.3389/fchem.2025.1537877

**Published:** 2025-03-07

**Authors:** Amine Elbouzidi, Mohamed Taibi, Abdellah Baraich, Mounir Haddou, Ramzi A. Mothana, Sami A. Alsufyani, Hany W. Darwish, Roland Molinié, Jean-Xavier Fontaine, Ophélie Fliniaux, François Mesnard, Mohamed Addi

**Affiliations:** ^1^ Laboratoire d’Amélioration des Productions Agricoles, Biotechnologie et Environnement (LAPABE), Faculté des Sciences, Université Mohammed Premier, Oujda, Morocco; ^2^ Laboratory of Bioresources, Biotechnology, Ethnopharmacology and Health, Faculty of Sciences, Mohammed First University, Oujda, Morocco; ^3^ Department of Pharmacognosy, College of Pharmacy, King Saud University, Riyadh, Saudi Arabia; ^4^ Department of Pharmaceutical Chemistry, College of Pharmacy, King Saud University, Riyadh, Saudi Arabia; ^5^ BIOPI-BioEcoAgro UMRT 1158 INRAE Université de Picardie Jules Verne, Amiens, France

**Keywords:** elicitation, phenolics and flavonoids production, chitosan, salicylic acid, antioxidant enzymes, antioxidant activity, anti-tyrosinase, anti-elastase

## Abstract

This research explores the effects of chitosan (CHT) and salicylic acid (SA) as elicitors on the production of phenolic and flavonoid compounds in *Pelargonium graveolens* Hort. Callus cultures on solid media, aiming to enhance antioxidant, anti-tyrosinase, and anti-elastase properties. Calli were treated with various concentrations of CHT (25, 50, 75, and 100 mg/mL) and SA (25, 50, 75, and 100 µM), and their phytochemical profiles were examined through LC-MS/MS analysis. The findings indicated that salicylic acid (SA) and chitosan (CHT) notably enhanced the levels of total phenolic content (TPC) and total flavonoid content (TFC). The greatest increase in TPC was seen in cultures treated with 25 µM of salicylic acid (SA_2_), recording 336.80 ± 8.35 mg/100 g dry weight (DW), and in cultures treated with 100 mg/mL of chitosan (CHT_5_), which showed 325.74 ± 7.81 mg/100 g DW. Among individual phenolics, kaempferol showed a remarkable increase under SA_2_ (192.82 ± 17.99 mg/100 g DW) compared to the control (103.68 ± 5.00 mg/100 g DW), and CHT_5_ treatment (119.68 ± 12.01 mg/100 g DW). Additionally, rutin accumulation peaked at 30.64 ± 3.00 mg/100 g DW under SA_2_ treatment. Antioxidant activities, measured by DPPH and TAC assays, were also enhanced, with SA_2_ and CHT_5_ treatments showing significant improvement over the control. The SA_2_-elicited cultures exhibited superior anti-tyrosinase and anti-elastase activities, with IC_50_ values of 51.43 ± 1.31 μg/mL, 35.42 ± 4.42 μg/mL, and 31.84 ± 0.60 μg/mL, respectively. These findings suggest that elicitors effectively boost the bioactive compound production in *P. graveolens* calli, and subsequently the biological activity, highlighting their potential in developing natural skincare products with antioxidant and anti-aging benefits.

## 1 Introduction

Plants, known for their wide range of chemical substances, functioning as the main sources of both primary metabolites and specialized secondary metabolites (PSMs) that are utilized in the pharmaceutical, food, and cosmetic industrial sectors (Yeshi et al., 2022). The cosmetic industry, in particular, has ramped up its pursuit of bioactive molecules with promising health benefits, mostly extracting these substances from herbal and medicinal plants (Kessler and Kalske, 2018). This pursuit has popularized the term “cosmeceutical,” which describes products that blend cosmetic appeal with pharmaceutical benefits to address skin conditions, integrating both aesthetic and therapeutic qualities (Marchev et al., 2020; Gupta et al., 2010). The very compounds that help plants withstand environmental challenges like salinity, UV exposure, drought, and extreme temperatures also safeguard human skin by triggering key protective signaling pathways shared among eukaryotic cells (Marchev et al., 2020; Gupta et al., 2010). Cosmeceuticals are formulated using a diverse array of bioactive phytochemicals, encompassing phenylpropanoids and their derivatives, alongside terpenes, including isoprenoids and terpenoids, as well as nitrogen-containing compounds like alkaloids and heterocyclic aromatics.

Key bioactive components, including vitamins, essential oils, amino acids, peptides, and sugars, play a crucial role in modulating specific signaling pathways within human skin cells. This modulation enhances the expression of genes linked to cellular aging and strengthens the skin’s resilience against various environmental and biological stressors (Wawrosch and Zotchev, 2021). Phytochemicals with antioxidant, anti-aging, antimicrobial, anti-inflammatory, anti-wrinkling, skin-whitening, and photoprotective properties are particularly valued in the cosmetics industry for their broad-spectrum efficacy in skincare applications (Saraiva et al., 2023). Beyond their cosmetic uses, these compounds demonstrate diverse therapeutic benefits. Flavonoids such as kaempferol and its derivatives exhibit strong antioxidant, anti-inflammatory, and anticancer activities, while isomers like isocoumarins are notable for their neuroprotective and antimicrobial properties (Ramanan et al., 2016; Sudarshan et al., 2019). Their therapeutic potential extends to managing chronic conditions, including neurodegenerative diseases and cancers, through the regulation of oxidative stress and inflammatory pathways (Ramanan et al., 2016; Sudarshan et al., 2019). These multifunctional properties underscore their versatility and importance in pharmaceuticals, nutraceuticals, and cosmeceuticals. Incorporating such bioactive compounds into skincare formulations enhances their functional efficacy and expands their potential applications, offering comprehensive health benefits.

A major part of pharmaceutical-grade secondary metabolites are derived from either wild-harvested or cultivated plants (Rungsung et al., 2015). Although chemical synthesis of these compounds has been extensively explored, it frequently remains economically unfeasible (Jansen and Shenvi, 2014). In this context, plant cell culture technology emerges as a promising alternative, offering significant advantages over conventional field cultivation and synthetic production. This approach is particularly valuable for obtaining metabolites derived from slow-growing plant species or those that are challenging to reproduce through chemical synthesis (Zhao and Verpoorte, 2007; Zárate and Verpoorte, 2007).

Plants utilize various defense mechanisms to resist pathogen invasion, including both pre-existing (constitutive) and inducible defenses, which are activated by specific chemical substances known as “elicitors” (Hammond-Kosack, 2000). The term “elicitor” originally referred to molecules that induce phytoalexin formation but now encompasses any substance that stimulates plant defenses (Eder and Cosio, 1994; Ramachandra Rao et al., 2002). These elicitors can originate from both biotic and abiotic sources and function at low concentrations as signaling molecules to initiate defense mechanisms, including the production of reactive oxygen species (ROS), the hypersensitive response, and phytoalexin synthesis, which possess antibacterial properties (Montesano et al., 2003; Boller, 1995). Activation of phytoalexin biosynthesis has become crucial in biotechnological strategies to enhance secondary metabolite synthesis.

PSMs, as Polyphenols, are increasingly recognized for their potential as cosmeceuticals due to their robust antioxidant properties and multiple skin health benefits (Humbal and Pathak, 2023; Ahmed et al., 2015). These compounds are particularly valued for their anti-tyrosinase and anti-elastase activities, making them effective in combating hyperpigmentation and preserving skin elasticity, respectively. Anti-tyrosinase activity helps reduce melanin synthesis, which can lead to a more even skin tone and reduced appearance of age spots. Anti-elastase activity, on the other hand, inhibits the breakdown of elastin, thereby aiding in maintaining skin’s structural integrity and elasticity. Furthermore, polyphenols exhibit natural sun protection factor (SPF) properties by absorbing UV radiation and neutralizing free radicals generated by sun exposure, thereby providing a layer of protection against photoaging and sunburn. As a result, these multifunctional properties make polyphenols a highly sought-after ingredient in the formulation of skincare products aiming to offer antioxidant, anti-aging, and photoprotective benefits. However, the production of these metabolites in plants is typically low, influenced by the plant’s physiological and developmental stages and environmental conditions (Bourgaud et al., 2001).

*Pelargonium graveolens* Hort., widely recognized as the “rose-scented geranium,” plays a significant role in various industries including cosmetics, aromatherapy, and food enhancement (Ćavar et al., 2012). Originating from South Africa, this perennial herb is valued for its visually appealing, lobed leaves and its pleasantly fragrant, rose-scented flowers. It has a long history of use in traditional medicine for the treatment of numerous health issues (Gomes et al., 2004). The medicinal benefits of *P. graveolens* are well-supported by research, with studies indicating its capabilities as an antioxidant (Lohani et al., 2019), and its anti-inflammatory and analgesic effects (Lalli et al., 2008). Additionally, it has been shown to possess strong anti-parasitic properties (Cock et al., 2018), and is effective in combating tuberculosis (Kardan-Yamchi et al., 2020). The herb’s antibacterial effects have been extensively studied, showing efficacy against a variety of pathogens (Lalli et al., 2008; Malik et al., 2011; Hsouna and Hamdi, 2012; Boukhatem et al., 2013; Singh et al., 2008). Despite its therapeutic potential, traditional farming techniques often do not optimize the production of these valuable bioactive compounds, leading to increased interest in alternative cultivation approaches such as plant cell cultures, which offer a sustainable and controlled method of production.

In the present research, chitosan (CHT), which is a natural derivative of chitin sourced from the shells of crustaceans, is investigated for its potential as a biotic elicitor in plant cell cultures. This compound is known for activating plant defense mechanisms and stimulating the synthesis of secondary metabolites (Golkar et al., 2019). Simultaneously, salicylic acid (SA), a phytohormone, is examined for its role as an abiotic elicitor. SA is recognized for bolstering plant defenses and increasing the production of bioactive compounds by influencing morphological, physiological, and biochemical processes in plants (Golkar et al., 2019). This dual approach leverages both biotic and abiotic elicitors to enhance the overall yield and quality of bioactive substances in plant cell culture systems.

In our previous research, we investigated the effect of chitosan and jasmonic acid as elicitors on *P. graveolens* suspension cultures (Elbouzidi et al., 2024a). While, the current study, is the first of its kind on the elicitation of *P. graveolens* callus cultures with chitosan and salicylic acid. The research aims to investigate and boost the production of bioactive compounds in *P. graveolens* Hort. Calli cultures using these elicitors, focusing on the impact of stressors on growth, polyphenol and flavonoid accumulation, antioxidant enzymatic defense responses, phytochemical profile changes through LC-MS/MS analysis, and evaluating the cosmeceutical properties of the elicited cultures, including antioxidant, anti-tyrosinase, anti-elastase activities, and sun protection factor assessment.

## 2 Materials and methods

### 2.1 Seed germination and callus establishement

Seeds of *P. graveolens* were obtained from Clorofila E-nursery located in Casablanca, Morocco. The seeds underwent a sterilization process based on a modified version of the protocol by Elbouzidi et al. (2024a). The stages involved in seed sterilization, germination, and callogenesis are detailed in Table 1, which provides a concise overview of the culture conditions used in the experimental procedures.

**TABLE 1:** Experimental procedures for seed sterilization, germination and callogenesis from leaf segments.

Stage	Medium components	Temperature	Photoperiod	Light intensity
Seed Sterilization	Washed with sterile water, 10% Sodium Hypochlorite (30 s), 70% ethanol (2 min)	N/A	N/A	N/A
Seed Germination	MS Basal Medium (Murashige and Skoog, 1962): 3% Sucrose, 0.8% Agar, pH 5.5–5.7	25°C ± 1°C	16 h light/8 h dark	40 μmol·m^−2^ ·s^−1^
Callogenesis	1 cm^2^ leaf segments from three-week-old seedlings were cultured on MS medium (Murashige and Skoog, 1962): 3% Sucrose, 0.8% Agar, 4.4 μM Thidiazuron (TDZ), pH 5.5–5.7	25°C ± 1°C	16 h light/8 h dark	40 μmol·m^−2^·s^−1^

### 2.2 Callus-induction and elicitors application

#### 2.2.1 Preparation of elicitors

For the elicitation process, chitosan (C_611_NO_4_, deacetylation degree 70%–85%) obtained from Merck Chemicals (Saint-Quentin Fallavier, France) and salicylic acid (S7401, Sigma-Aldrich, St. Louis, Missouri) were utilized. Both Chitosan and Salicylic acid were prepared by dissolving each in different solvents to ensure proper dissolution. Chitosan was dissolved in 0.1% acetic acid and continuously stirred at 50°C for 5 hours for complete dissolution. In contrast, Salicylic acid was prepared as a 1 mM solution by dissolving it in distilled water and stirring for 2 hours to achieve uniform solubility. Both elicitors solutions were pH-adjusted to 5.5–5.7, and were introduced into MS-based culture media at varying concentrations. Chitosan was added at concentrations of 5, 25, 50, 75, and 100 mg/L, while salicylic acid was incorporated at 5, 25, 50, 75, and 100 µM. For each treatment, 1 mL of the respective elicitor solution was mixed with the culture medium. As a reference, a control medium was prepared by adding 1 mL of fresh MS medium devoid of any elicitors, providing a baseline for comparison.

#### 2.2.2 Calli elicitation

To initiate elicitation, leaf-derived calli weighing 0.5 g (fresh weight) were transferred onto freshly prepared solid Murashige and Skoog (MS) medium (Murashige and Skoog, 1962).

In each 90 mm Petri dish, 15 mL of MS medium was used, solidified with agar and enhanced with 4.45 µM TDZ, 30 g/L sucrose, along with various concentrations ranging from 5 to 100 mg/L of chitosan and 5–100 µM of jasmonic acid. The cultures were kept in a stable growth chamber at 25°C ± 1°C. The environmental conditions for incubation consisted of a light cycle of 16 h followed by a dark period of 8 h, and a maintained light intensity between 40 and 50 μmol·m^−2^ ·s^−1^ to promote ideal growth and elicitation.

### 2.3 Sample extraction

Calli extraction was conducted following the protocol established by Ahmad et al. (1998) (Ahmad et al., 1998). To prepare methanolic extracts, 10 g of dried calli were finely powdered and thoroughly mixed with 100 mL of 80% methanol. The mixture was subjected to sonication for 10 min in an ultrasonic bath (Toshiba, Japan) to facilitate the release of compounds. Following sonication, the sample was vigorously vortexed for 20 min to ensure complete mixing. To optimize the extraction yield, this procedure was repeated three times. After extraction, the combined samples were centrifuged at 13,000 rpm for 15 min, and the clear supernatants were then carefully decanted. These supernatants were preserved at 4°C to ensure the stability of the compounds until needed for further analysis.

#### 2.3.1 Total Phenolic Contents

Folin-Ciocalteu’s method, as described by Slinkard and Singleton (1977) (Slinkard and Singleton, 1977), was slightly modified to determine total phenolic content, the detailed protocol is detailed in (Elbouzidi et al., 2024b). Total phenolic production (TPP) was expressed in mg GAE/100 g DW was calculated using a specific formula.

$$\text{Total Phenolic Production }\left(\text{mg}/L\right)=\text{DW }\left(g/L\right)\times \text{TPC }\left(\text{mg}/g\right)$$

#### 2.3.2 Total Flavonoid Contents

The total flavonoid content (TFC) was determined using the aluminum chloride method as described by Chang et al. (2002) (Chang et al., 2002), with the full protocol provided in (Elbouzidi et al., 2024b). Total flavonoid production (TFP) was calculated in milligrams of quercetin equivalents per liter (mg QE/L) using a specific formula.

$$\text{Total Flavonoids Production }\left(\text{mg}/L\right)=\text{DW }\left(g/L\right)\times \text{TFC }\left(\text{mg}/g\right)$$

### 2.4 Quantification of phenolic compounds using LC-MS/MS

Ultra-Performance Liquid Chromatography-Mass Spectrometry (UPLC-MS) analyses were conducted using an ACQUITY UPLC I-Class system paired with a Vion IMS QTof hybrid mass spectrometer (Waters, Manchester, UK), which features an electrospray ionization (ESI) source. A 1 µL aliquot of each sample was injected into a Kinetex Biphenyl column (100 × 2.1 mm, 1.7 µm; Phenomenex, Torrance, CA, United States), held at a constant temperature of 55°C. Chromatographic separation was achieved with a mobile phase flow rate of 0.50 mL/min, utilizing a gradient elution from 0.1% formic acid in water (A) to 0.1% formic acid in methanol (B) as follows: 80:20 at 0 min and 0.5 min, 40:60 at 5 min, 10:90 at 6 and 7 min, then returning to 80:20 at 7.5 min and held until 10 min.

The ESI source was set to operate in negative ionization mode, with a capillary voltage of 2.5 kV and a sampling cone voltage of 20 V. Source temperature was maintained at 120°C, while the desolvation temperature was 450°C. Nitrogen served as both the desolvation gas (800 L/h), and cone gas (50 L/h). Data were processed using UNIFI software, with UV quantifications performed automatically through the Quantify UV methods. Mass spectrometric analysis was based on retention time (RT), accurate mass-to-charge ratio (*m/z*), and molecular formula.

UV detection was conducted at 280 nm, a wavelength corresponding to the maximum absorbance of phenolic compounds. Calibration curves for quantification were prepared with standard solutions in methanol ranging from 0.001 to 1 mg/mL. The results, detailed in Supplementary Figures S1, S2, and Supplementary Tables S1, S2, were derived using 7-point calibration curves, all of which exhibited coefficients of determination (R^2^) exceeding 0.90. Each sample was analyzed in triplicate, with outcomes expressed as milligrams per 100 g of dry weight. The limit of detection (LOD) and the limit of quantification (LOQ) are both measures used to determine the smallest concentration of a substance that can be reliably detected, and measured, respectively, following the formulas provided by Loukili et al. (2021).

### 2.5 Antioxidant enzymes activity

#### 2.5.1 Peroxidase (POD) activity

The measurement of peroxidase activity utilized a protocol adapted from references (Khan et al., 2019), and (Lagrimini, 1980). For the initial sample preparation, 100 mg of fresh tissue was blended with 1 mL of 50 mM potassium phosphate buffer (pH 7) that included 1% polyvinylpyrrolidone (PVP). This mixture was then centrifuged at 15,000 rpm for 30 min, and the clear supernatant was extracted for analysis. The assay mixture for determining POD activity contained 40 µL of potassium phosphate buffer, 20 µL of guaiacol at 100 mM concentration, 20 µL of the sample extract, 100 µL of distilled water, and 20 µL of 27.5 mM hydrogen peroxide. A control sample was prepared without the sample extract, keeping all other components constant. Absorbance changes were measured spectrophotometrically using an extinction coefficient of 6.39 mM^−1^·cm^−1^. Enzyme activity, expressed as nM/min/mg of fresh weight, was calculated based on absorbance and a path length of 0.25 cm, where A is absorbance, E is the extinction coefficient (6.39 mM^−1^·cm^−1^), L is the path length (0.25 cm), and C is the concentration (nM/min/mg of fresh weight).

A=E .L .C

#### 2.5.2 Superoxide dismutase (SOD) activity

The assay for superoxide dismutase activity followed a modified protocol from Khan et al. (2019). The reaction setup included 20 µL of 1 mM EDTA, 20 µL of 130 mM methionine, 20 µL of 0.75 mM nitro-blue tetrazolium (NBT), 78 µL of 50 mM phosphate buffer (pH 7), 2 µL of 0.02 mM riboflavin, and 60 µL of freshly prepared sample extract. A control was similarly prepared, omitting the sample extract. These mixtures were then exposed to fluorescent light for a duration of 7 min, after which the absorbance was measured at 660 nm using a Thermo Scientific Multiskan GO microplate reader. The enzymatic activity was determined using a calculation method analogous to that used for peroxidase activity.

### 2.6 Antioxidant activity

#### 2.6.1 DPPH assay

The assessment of antioxidant activity was conducted using an adapted DPPH assay (Elbouzidi et al., 2024b; Taibi et al., 2023). A solution of DPPH was made by dissolving 2 mg of the substance in 100 mL of methanol. In each assay, 2.5 mL of this DPPH solution was combined with a test sample and the total volume was brought up to 3 mL. The mixture was then allowed to incubate at room temperature for 30 min, followed by the measurement of absorbance at 517 nm using a blank as a reference. From this, the percentage of DPPH free radical scavenging activity (FRSA) was calculated.

$$\text{FRSA }\left(\%\right)=\left[\left(\frac{{A\;}_{\text{blank}}-{A\;}_{\text{sample}}}{{A\;}_{\text{blank}}}\right)\right]\times 100$$

The control reaction, containing all reagents without extract, was used to measure the absorbance, designated as A_blank_. The absorbance for each extract concentration was recorded as A_sample_. To determine the IC_50_ value, a graph was plotted showing the percentage inhibition against the extract concentrations. Ascorbic acid served as the reference standard.

#### 2.6.2 TAC assay

The assessment of total antioxidant capacity utilized the phosphor-molybdenum assay, following the protocol outlined by Chaudhary et al. (2015). The procedure involved mixing 0.1 mL of either a standard or an extract with 0.3 mL of a reagent mixture composed of 0.6 M sulfuric acid, 28 mM sodium phosphate, and 4 mM ammonium molybdate, followed by incubation at 95°C for 90 min. Post-incubation, the samples were allowed to reach room temperature before the absorbance at 695 nm was recorded. A blank solution, which included all reagents with no test sample, was also prepared. Ascorbic acid was employed to generate a standard curve, and results were expressed as ascorbic acid equivalents, according to Prieto et al. (1999). Replicates of each experiment were performed three times.

### 2.7 Anti-tyrosinase, and anti-elastase activity

#### 2.7.1 Anti-tyrosinase activity

The evaluation of tyrosinase inhibition was conducted according to the protocol established by Momtaz et al. (2008), utilizing substrates such as L-3,4-dihydroxyphenylalanine (L-DOPA) and L-tyrosine. Samples were initially dissolved in DMSO and then diluted in a 50 mM potassium phosphate buffer at pH 6.5. The testing occurred in a 96-well plate where absorbance was recorded using a Multiskan FC microplate reader. In the assay, 25 µL of each sample was combined with 30 µL of tyrosinase solution (333 units/mL in phosphate buffer, pH 6.5). After resting at room temperature for 5 min, 110 µL of either a 2 mM L-tyrosine or 12 mM L-DOPA substrate solution was added. The reaction was allowed to proceed for 30 additional minutes. Kojic acid served as the positive control. Blanks were prepared containing all components except for the substrates (L-tyrosine or L-DOPA). Measurements were made at an absorbance of 492 nm to determine the percentage inhibition of tyrosinase, calculated using the following equation, (Elbouzidi et al., 2024c):

$$\text{Tyrosinase inhibition }\left(\%\right)=\left(\frac{A_{\text{control}}-A_{\text{sample}}}{A_{\text{control}}}\right)\times 100$$

Where A_control_ and A_sample_ denote the absorbance values of the blank and the test reaction mixture (containing either the sample or kojic acid), respectively. Tyrosinase inhibition was evaluated across concentrations ranging from 10 to 500 μg/mL, and IC_50_ values were determined. Kojic acid (Sigma, St. Louis, MO, United States) served as the positive control in this study.

#### 2.7.2 Elastase activity

The anti-elastase activity was assessed spectrophotometrically following the procedure outlined by Taibi et al. (2024), with minor adjustments. A stock solution of elastase from porcine pancreas was prepared at a concentration of 3.33 mg/mL in sterile water. The substrate, N-succinyl-Ala–Ala–Ala–p-nitroanilide (AAAPVN), was dissolved in Tris–HCl buffer (pH 8) at a concentration of 1.6 mM. Test samples at concentrations ranging from 1 to 500 μg/mL were incubated with the elastase solution in the buffer at room temperature for 15 min. The reaction was initiated by adding the synthetic substrate. Oleanolic acid served as the positive control, while water was used as the negative control. Absorbance was recorded at 400 nm using a microplate reader. Elastase inhibition (%) was calculated using the following formula:

$$Elastase\;inhibition\;\left(\%\right)=\left[1-\left(\frac{S}{C}\right)\right]\times 100$$

where “S” represents the corrected absorbance of the test samples, and “C” represents the corrected absorbance of the controls (without sample). The IC_50_ value was determined from dose-response curves generated using GraphPad Prism software version 8.0 (San Diego, CA, United States).

### 2.8 Sun protection factor

The sun protection factor (SPF) for calli extracts was assessed via the UV absorbance technique, adhering to the method outlined by Mansur et al. (1986) (Mansur et al., 1986b). Absorbance levels of the calli extracts, prepared at a concentration of 0.1 mg/mL, were measured between 290 and 320 nm at 5 nm intervals, with each wavelength undergoing three absorbance readings. The SPF values were derived using Equation 1, and the percentage of UVB radiation blocked was determined using formula 2.

$$\text{SPF}=\text{CF}\times \sum_{290}^{320}\left[EE\left(\lambda \right)\times I\left(\lambda \right)\times Abs\;\left(\lambda \right)\right]$$ (1)

$$\%\text{ Blocked UVB}=\left[1-\left(\frac{1}{SPF}\right)\right]\times 100$$ (2)

Here, CF represents the correction factor, set at 10, EE(λ) denotes the erythemogenic effect of radiation at wavelength λ, I(λ) refers to the solar intensity spectrum, and Abs(λ) indicates the spectrophotometric absorbance at wavelength λ. The constants for EE(λ) × I(λ) was established by Sayre et al. (1979), and are listed in Table 2.

**TABLE 2:** Relationship between erythemogenic effect and radiation intensity.

Wavelength (nm)	EE X I (Normalized)
290	0.0150
295	0.0817
300	0.2874
305	0.3278
310	0.1864
315	0.0837
320	0.0180
Total	1

### 2.9 Statistical analysis

Every experiment was performed with at least three replicates, and results are expressed as the mean ± standard deviation (SD) based on these replicates. Graphs displaying the mean values and SD were created using GraphPad Prism software (GraphPad Software, San Diego, CA, United States). Statistical analyses, including the calculation of means, standard deviations, and analysis of variance, were conducted using SPSS v19.0 (IBM Corp., Armonk, NY, United States), with statistical significance established at *p* < 0.05.

## 3 Results

### 3.1 Preliminary optimization

**FIGURE 1:**
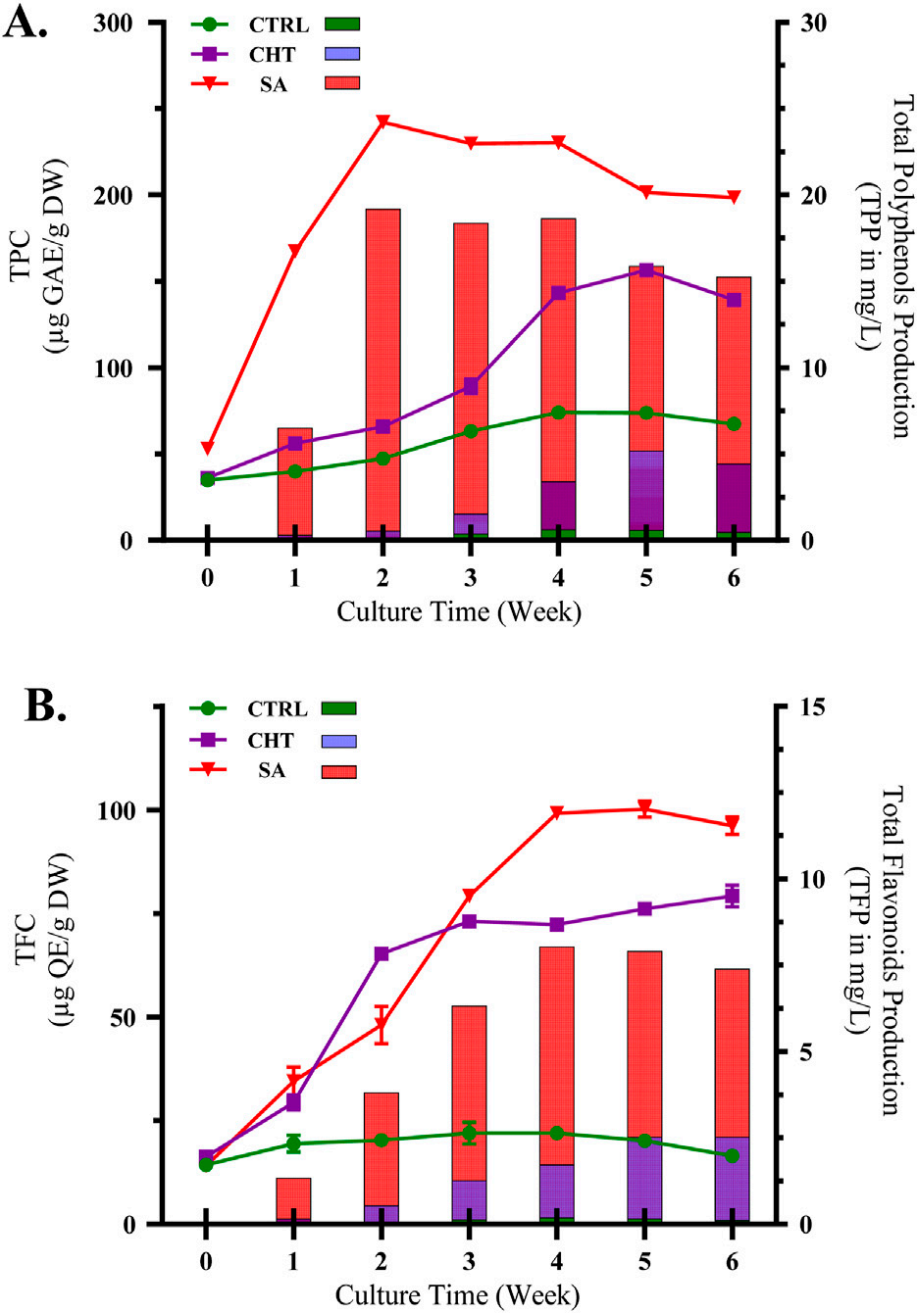
Accumulation of total polyphenols **(A)** and flavonoids **(B)** in *P. graveolens* calli cultures elicited with chitosan (CHT_5_, 100 mg/mL) and salicylic acid (SA_2_, 25 µm) over different growth periods. Data are expressed as means ± standard deviations from triplicate experiments.

The study explored the effects of elicitors like chitosan (CHT) and salicylic acid (SA) on the production of total polyphenols (TPC) and flavonoids (TFC) in callus cultures of *P. graveolens* to evaluate their role in enhancing secondary metabolite synthesis (Table 3). According to Figure 1A, TPC concentrations in the control group (CTRL) were consistently low throughout the experiment, indicating a restrained natural synthesis of polyphenols in the absence of elicitors. In contrast, treatment with CHT5 at a concentration of 100 mg/mL resulted in a notable rise in TPC, reaching peak values between the fourth and fifth weeks, ranging from 143.20 ± 2.38 to 156.53 ± 0.41 µg GAE/g DW, before slightly decreasing to 139.30 ± 3.50 µg GAE/g DW. This pattern is consistent with previous findings that chitosan, a biopolymer originating from chitin, stimulates the production of phenolic compounds by activating defense mechanisms and enhancing the activity of enzymes such as phenylalanine ammonia-lyase (PAL) (Ferri and Tassoni, 2011; Chalchat et al., 1998; Gai et al., 2019).

**TABLE 3:** Elicitors impact on growth parameters, biomass, secondary metabolites, mainly, polyphenols, and flavonoids. DW refers to dry weight.

Elicitor	Concentrations	Initiation day	Callus characteristics		Maximum biomass (g/100 mL)		Optimum values	
			Color	Texture			TPC (µg GAE/g DW)	TFC (µg QE/g DW)
					FW	DW		
Control	-	**4th**	**G**	**F**	**8.69 ± 0.36** ^**b**^	**0.85 ± 0.09** ^**a**^	**74.06 ± 0.52** ^**b**^	**22.01 ± 2.65** ^**c**^
CHT (mg/mL)	5	6th	LG	C	8.82 ± 0.18^bc^	0.75 ± 0.07^a^	81.02 ± 2.31^b^	19.07 ± 1.02^ab^
	25	8th	GB	C	10.72 ± 0.65^cd^	0.65 ± 0.09^a^	76.31 ± 0.30^b^	16.56 ± 2.45^a^
	50	3rd	LB	C	9.63 ± 0.65^bc^	0.85 ± 0.11^a^	78.23 ± 0.47^b^	25.98 ± 0.05^c^
	75	6th	DG	F	14.92 ± 1.52^e^	1.04 ± 0.36^a^	139.98 ± 0.21^d^	56.10 ± 0.46^e^
	100	**2nd**	**G**	**F**	**19.27 ± 1.08** ^**f**^	**3.32 ± 0.19** ^**b**^	**156.53 ± 0.41** ^**e**^	**73.09 ± 0.08** ^**g**^
SA (µM)	5	4th	LG	C	9.82 ± 0.02^bc^	1.09 ± 0.02^a^	63.12 ± 1.05^a^	17.06 ± 0.07^a^
	25	**5th**	**LB**	**C**	**31.18 ± 0.51** ^**i**^	**7.93 ± 0.29** ^**d**^	**242.09 ± 0.18** ^**f**^	**99.18 ± 0.04** ^**i**^
	50	3rd	DG	C	25.62 ± 0.51^h^	5.05 ± 0.99^c^	136.41 ± 4.62^d^	61.28 ± 0.95^f^
	75	9th	GB	C	11.83 ± 0.08^d^	1.21 ± 0.07^a^	120.18 ± 3.98^c^	51.19 ± 0.67^d^
	100	8th	DB	C	6.09 ± 0.23^a^	0.76 ± 0.02^a^	159.09 ± 5.01^e^	78.11 ± 1.93^h^

GB to Green Brown; DG to Dark Green; DB to Dark Brown; LG to Light Green; LB to Light Brown; G to Greenish. Texture is indicated as C: Compact and F: Friable. TPC represents Total Phenolic Contents, and TFC stands for Total Flavonoid Contents. The data are presented as means ± standard error, with different letters in each column denoting statistically significant differences based on the Tukey test (p ≤ 0.05). Bolded values highlight the optimum results.

In contrast, treatment with SA_2_ at 25 µM resulted in a significantly stronger increase in TPC, with a swift elevation observed within the initial 2 weeks, reaching a peak in the third week at 242.09 ± 0.18 µg GAE/g DW, and sustaining elevated levels through the sixth week at 198.49 ± 1.50 µg GAE/g DW. Salicylic acid, recognized as a signaling molecule, initiates systemic acquired resistance in plants, thereby activating genes associated with phenolic biosynthesis (Ahmad et al., 2019; Singh and Dwivedi, 2018). The continuous high levels of polyphenols in cultures treated with SA underscore its effectiveness in enhancing secondary metabolite pathways, likely through its influence on the regulation of stress-responsive gene expression and the activity of enzymes involved in the synthesis of phenolic compounds.

The impact of SA elicitation on TPP is significantly more pronounced, with an immediate and sharp increase in polyphenol production beginning in the first week, peaking by the third week, and sustaining high levels through the sixth week. This marked and consistent rise in TPP indicates that SA is an effective elicitor, strongly enhancing the biosynthetic processes associated with polyphenol production. The ability of SA to initiate defense mechanisms likely boosts the activity of crucial enzymes within the phenylpropanoid pathway, leading to substantial increases in polyphenol levels. The pronounced elicitation effect of SA highlights its potential as a powerful agent for enhancing secondary metabolite production in plant cultures, thereby increasing the bioactive compound concentrations in *P. graveolens*. This detailed evaluation of both TPC and TPP illustrates the significant influence of elicitor dosage on polyphenol production, reflecting the overall effectiveness of the elicitation approach.

Likewise, the presence of both elicitors substantially increased the accumulation of TFC (Figure 1B) compared to the control group. The control exhibited only a modest rise in flavonoid levels, indicative of a limited natural stimulation of flavonoid biosynthesis. Treatment with chitosan prompted a significant increase in TFC, with a peak observed in the third week (73.09 ± 0.08 µg QE/g DW), and a slight further increase by the sixth week (79.21 ± 2.60 µg QE/g DW). The effect of chitosan on flavonoid synthesis is supported by its role in simulating biotic stress, which activates flavonoid biosynthetic pathways via signaling cascades (Li et al., 2017; Samari et al., 2020). Conversely, SA treatment resulted in the highest increase in flavonoid levels, peaking in the fourth week (99.18 ± 0.04 µg QE/g DW) and maintaining high levels until the study’s conclusion. This aligns with research indicating that salicylic acid not only boosts flavonoid biosynthesis but also regulates the antioxidant capabilities of plants through its influence on key enzymes such as chalcone synthase and flavonoid hydroxylase (Tahjib-Ul-Arif et al., 2018; Gacnik et al., 2021; Li et al., 2019).

These results highlight the varying effects of chitosan (CHT) and salicylic acid (SA) on secondary metabolite synthesis in *P. graveolens* calli, with SA showing greater effectiveness in boosting both total polyphenol content (TPC) and total flavonoid content (TFC). The significant increase in polyphenols and flavonoids due to these elicitors indicates that refining elicitation strategies using SA may be an effective method for augmenting the bioactive compound levels in medicinal plants, an aspect essential for their therapeutic uses (Mahajan et al., 2022). Moreover, the peak accumulation observed around weeks 3-4 suggests an optimal timeframe for the synthesis of secondary metabolites under these experimental conditions, shedding light on the dynamic nature of plant cell cultures’ responses to elicitation treatments.

### 3.2 Phenolic compounds quantification using LC-MS/MS

Plants generate a wide variety of plant secondary metabolites as a defense mechanism to various signaling molecules and stress factors. Under stress conditions, plants reallocate resources from primary metabolism, redirecting primary metabolites (PMs) towards the biosynthesis of secondary metabolites (SMs). These secondary compounds include phenolic compounds, flavonoids, tannins, and other specialized metabolites crucial for stress adaptation. This metabolic shift is often accompanied by an upregulation of antioxidant enzyme activities, enhancing the plant’s ability to combat oxidative damage and maintain cellular homeostasis (Humbal and Pathak, 2023).

**FIGURE 2:**
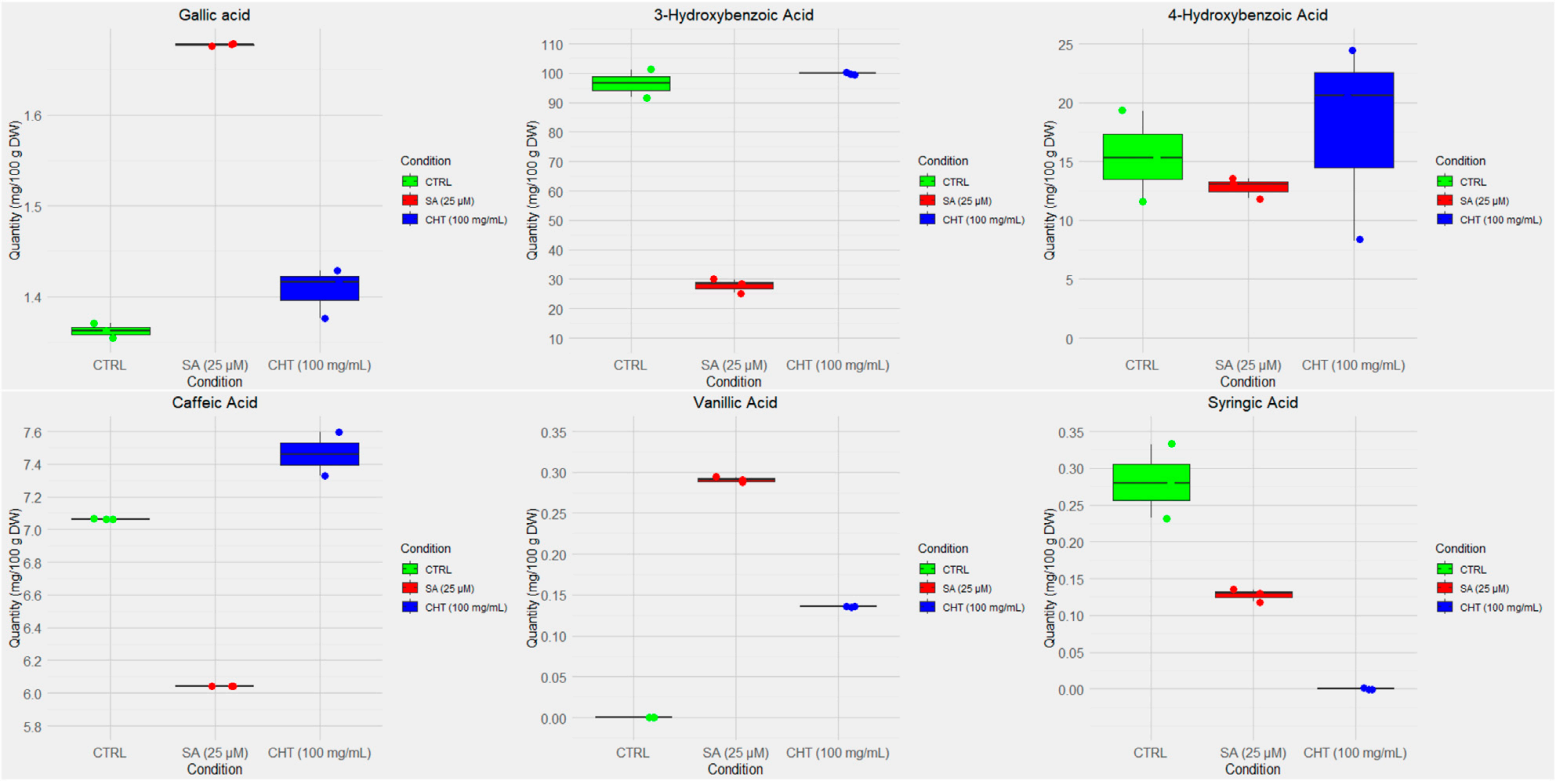
Box and whisker plots of the UV-quantified compounds in *P. graveolens* non-elicited cultures (control) and elicited cultures (CHT-elicited, and SA-elicited calli cultures).

**FIGURE 3:**
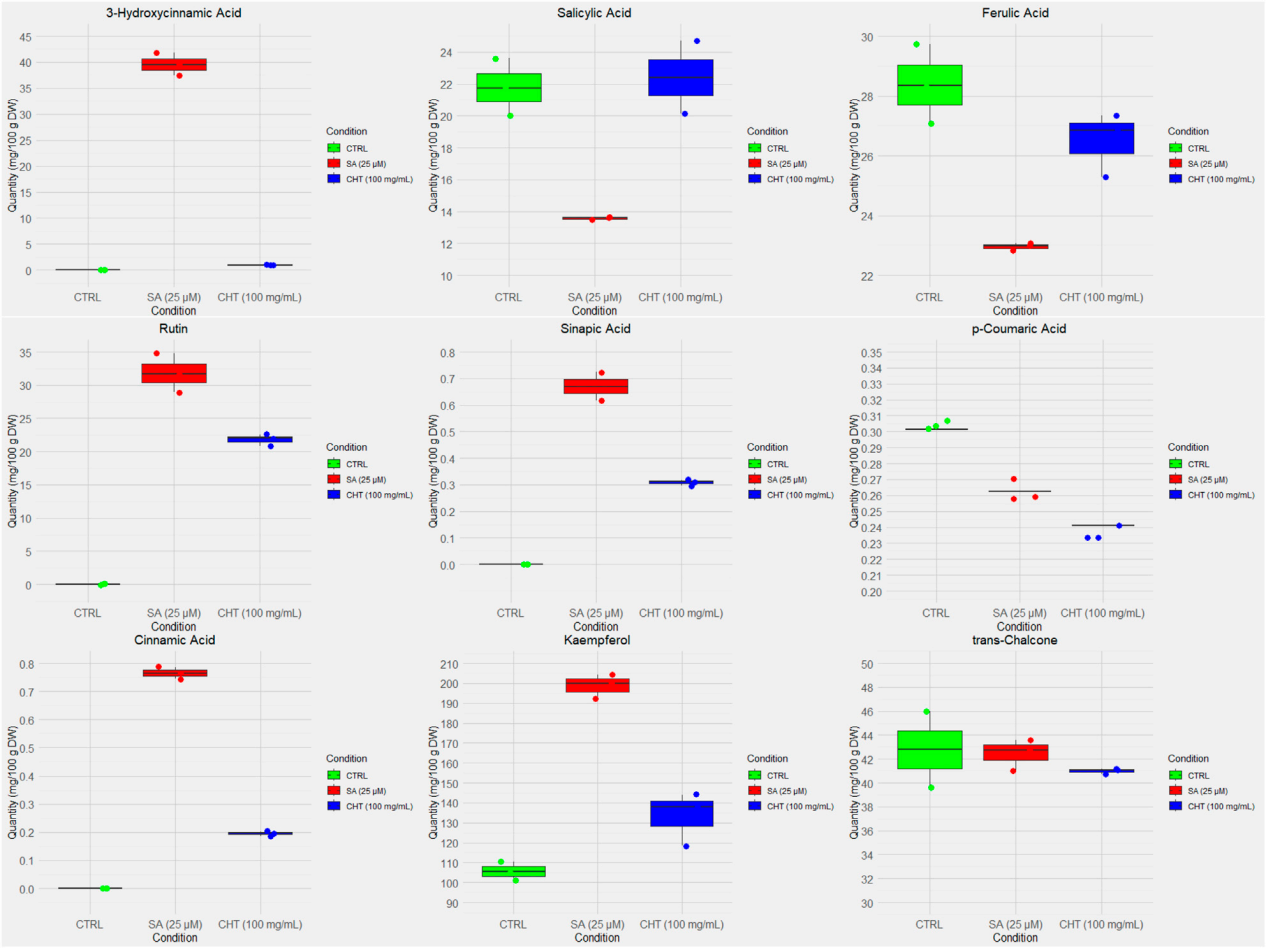
Cont.

The quantification of phenolic compounds in *P. graveolens* callus cultures elicited with chitosan (CHT_5_, 100 mg/mL) and salicylic acid (SA_2_, 25 µM) reveals distinct biosynthetic responses compared to the control (Figures 2, 3). Notably, Gallic acid levels showed a significant increase under SA_2_ treatment (1.6758 ± 0.0040 mg/100 g DW) compared to control (1.3591 ± 0.0085 mg/100 g DW, *p* < 0.01) and CHT_5_ (1.5320 ± 0.0071 mg/100 g DW, *p* < 0.05). This increase suggests that salicylic acid may modestly enhance the phenylpropanoid pathway. This observation aligns with reports suggesting that SA_2_ enhances the phenylpropanoid pathway, boosting the biosynthesis of hydroxybenzoic acid derivatives (Maurya et al., 2019). Conversely, 3-hydroxybenzoic acid, which was abundant in the control (97.4853 ± 10.1739 mg/100 g DW), decreased significantly under SA (25.9973 ± 6.0549 mg/100 g DW, *p* < 0.001), indicating a potential diversion of precursor metabolites to other phenolic pathways. Chitosan treatment maintained 3-hydroxybenzoic acid levels (99.8320 ± 0.0000 mg/100 g DW, *p* > 0.05) comparable to the control.

Flavonoid accumulation showed a distinct response to the elicitors. Rutin content was significantly higher under SA_2_ treatment (30.6360 ± 0.0023 mg/100 g DW) compared to both control (15.430 ± 0.0010 mg/100 g DW, *p* < 0.001) and CHT_5_ (21.1040 ± 0.0040 mg/100 g DW, *p* < 0.05), suggesting that SA more effectively stimulates flavonoid glycosylation (Golkar et al., 2019). Similarly, kaempferol levels increased significantly under SA_2_ (192.8160 ± 3.9977 mg/100 g DW) relative to control (103.6787 ± 2.0099 mg/100 g DW, *p* < 0.001) and CHT_5_ treatments (119.6773 ± 1.0129 mg/100 g DW, *p* < 0.05), indicating that SA strongly enhances flavonoid biosynthesis pathways. This highlights the elicitor’s role in selectively promoting flavonoid biosynthesis, possibly through the activation of glycosyltransferase enzymes that enhance flavonoid glycosylation (Golkar et al., 2019).

Interestingly, *trans*-chalcone, and 3-hydroxycinnamic acid showed minimal variation across treatments, with no statistically significant differences observed, suggesting that these metabolites are less responsive to the elicitors used in *P. graveolens* calli cultures.

**FIGURE 4:**
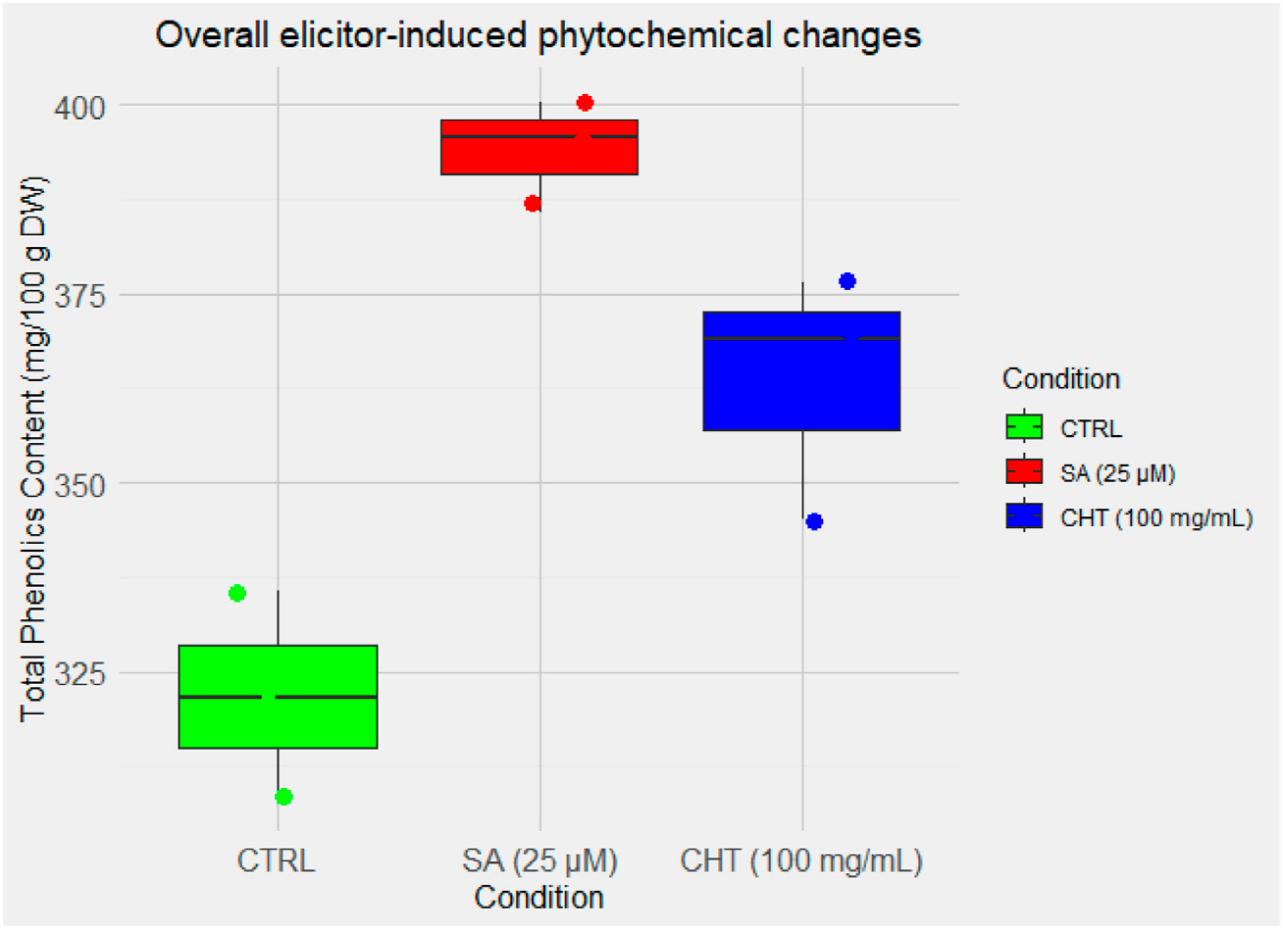
Overall elicitor-induced phytochemical changes in *P. graveolens* calli cultures.

The analysis of total phenolics content (TPC) revealed a general increase in phenolic accumulation in both elicited callus cultures. SA_2_ elicitation resulted in a slightly higher TPC (387.79 ± 19.79 mg/100 g DW) compared to CHT_5_ (346.64 ± 14.74 mg/100 g DW), with the difference being statistically significant (*p* < 0.05). Both treatments also demonstrated statistically significant increases compared to the control (316.42 ± 14.03 mg/100 g DW), with CHT_5_ significant at *p* < 0.05, and SA_2_ at *p* < 0.01 (Figure 4).

These findings are consistent with previous studies on *P. graveolens*, such as those by Elbouzidi et al. (2024d), which highlighted distinct metabolic responses in suspension cultures treated with chitosan and jasmonic acid (JA). In suspension cultures, phenolic acids like syringic and ferulic acids were significantly induced under JA, with ferulic acid levels reaching 1,562.87 ± 1.75 mg/100 g DW, a much higher response compared to the moderate levels observed in callus cultures. In contrast, this study demonstrated that chitosan elicitation in callus cultures specifically enhanced the biosynthesis of phenolics such as 3-hydroxybenzoic acid, *p*-coumaric acid, sinapic acid, and vanillic acid, with concentrations ranging from 0.2623 to 25.9973 mg/100 g DW. These responses were not observed in *P. graveolens* suspension cultures elicited with chitosan.

The observed enhancement of phenolic and flavonoid compound production in *P. graveolens* callus cultures treated with chitosan and salicylic acid can be attributed to their roles as elicitors in activating plant defense pathways. Chitosan, a biopolymer derived from chitin, is known to mimic biotic stress, leading to the activation of signal transduction pathways that upregulate phenylalanine ammonia-lyase (PAL) and other enzymes involved in the phenylpropanoid pathway (Xoca-Orozco et al., 2019). This results in increased biosynthesis of phenolics and flavonoids, which act as defensive secondary metabolites. Similarly, salicylic acid functions as a phytohormone, inducing systemic acquired resistance (SAR) and activating stress-responsive genes. These genes enhance the activity of enzymes like chalcone synthase and flavonoid hydroxylase, critical for flavonoid biosynthesis (Tripathi et al., 2019).

These differences may be attributed to the distinct nature of the two culture systems. Callus cultures on solid media experience limited nutrient and oxygen diffusion compared to liquid suspension cultures, which could influence the metabolic activity and elicitor responsiveness (Shukla et al., 2024). Solid media may promote localized metabolic responses due to the heterogeneous distribution of elicitors, whereas liquid cultures facilitate uniform elicitor exposure, potentially leading to stronger or more widespread metabolic induction (Cai et al., 2012). Additionally, the physiological state of cells differs: callus tissues are more differentiated than suspension cells, which may lead to variations in the regulation of secondary metabolite pathways. These findings highlight the critical role of culture conditions in modulating phenolic biosynthesis and emphasize the need to tailor elicitation strategies for specific culture systems to optimize secondary metabolite production. Such differences underscore the impact of culture conditions on metabolite biosynthesis. Solid media in callus cultures may limit nutrient and oxygen diffusion, promoting localized responses, while liquid suspension cultures offer uniform elicitor exposure, leading to broader metabolic induction (Fazili et al., 2022).

The findings highlight the need to tailor elicitation strategies to specific culture systems for optimizing secondary metabolite production. Future studies could focus on combining elicitors or adjusting their concentrations and exposure durations to maximize the yield of target compounds. Additionally, integrating transcriptomic and proteomic analyses could offer deeper insights into the regulatory mechanisms underlying these metabolic changes.

### 3.3 Antioxidant enzymes quantification

In this study, we investigated the enzymatic responses of *P. graveolens* callus cultures treated with chitosan (CHT) and salicylic acid (SA) at varying concentrations, focusing on the activities of peroxidase (POD) and superoxide dismutase (SOD) as biomarkers of oxidative stress and defensive metabolic activity. Elicitor treatments initiate a series of cellular events, starting with signal transduction at the plasma membrane surface, which subsequently leads to the production of reactive oxygen species (ROS) (Benhamou, 1996). This process activates the plant’s defense pathways and stimulates secondary metabolism by modulating the activity of key enzymes involved in the biosynthesis of secondary metabolites (Zhao et al., 2005).

The oxidative stress induced by elicitor exposure results in elevated ROS levels, which play a dual role: acting as signaling molecules for defense activation and, if unchecked, causing cellular damage. Plants counteract this imbalance through the upregulation of antioxidant enzymes, including superoxide dismutase (SOD), peroxidase (POD), ascorbate peroxidase (APX), and catalase (CAT), which collectively mitigate ROS-induced damage (Fujita and Hasanuzzaman, 2022). However, when the production of ROS exceeds the scavenging capacity of these enzymes, oxidative damage escalates, compromising cell viability and potentially leading to programmed cell death. These dynamic highlights the importance of balancing ROS generation and antioxidant responses for maintaining cellular integrity under elicitor-induced stress.

Superoxide dismutase (SOD) and peroxidase (POD) enzymes are isoforms with distinct physical, chemical, and structural characteristics, working in concert to preserve cellular redox balance and shield plants from damage caused by oxidative stress. SOD facilitates the dismutation of superoxide radicals into molecular oxygen and hydrogen peroxide, while POD reduces hydrogen peroxide by oxidizing various substrates, resulting in water and oxidized products.

**FIGURE 5:**
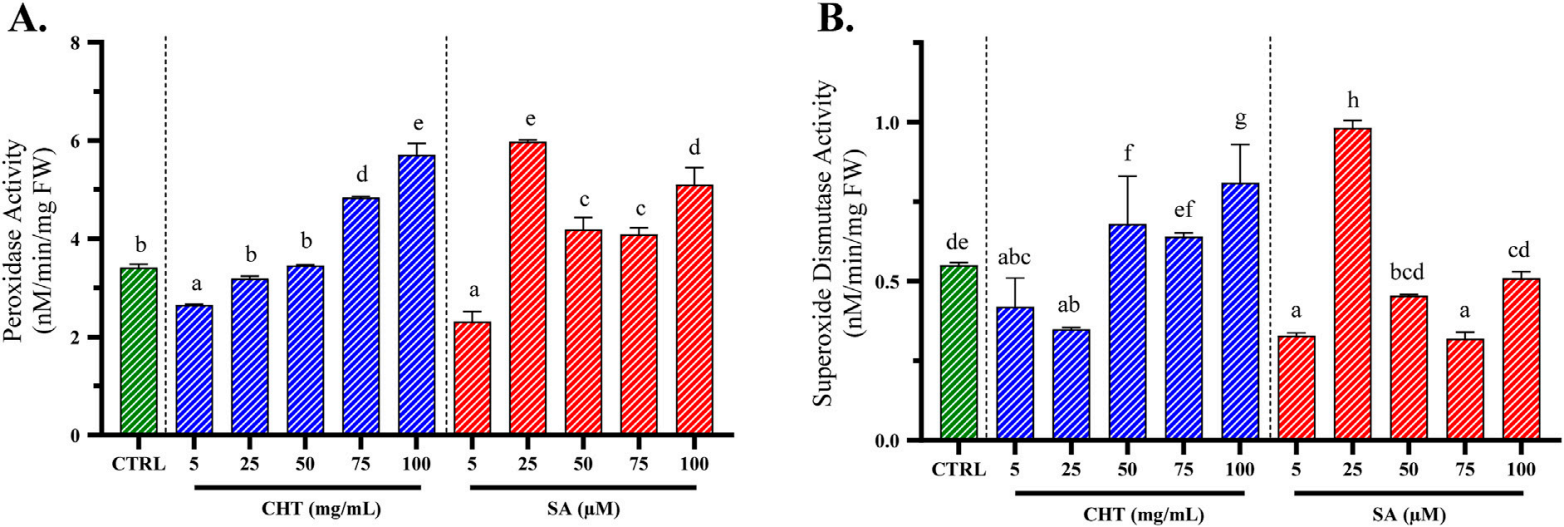
Peroxidase (POD; **(A)**) and superoxide dismutase (SOD; **(B)**) activities in *P. graveolens* suspension cultures treated with chitosan (CHT) and salicylic acid (SA) compared to the CTRL group (untreated calli). Values are presented as means ± standard error, with different letters denoting statistically significant differences based on the Tukey test (*p* ≤ 0.05).

In this study, the impact of chitosan (CHT) and salicylic acid (SA) on the activities of SOD and POD was evaluated in *P. graveolens* callus cultures. The findings revealed that the enzymatic activities of both SOD and POD increased proportionally with the concentration of elicitors applied (Khan et al., 2019; Liu et al., 2023; Ali et al., 2020; Al-Dhabaan et al., 2018). The results of these experiments are presented in Figure 5.

The influence of chitosan (CHT) on peroxidase (POD) activity was clearly dose-dependent, showing a progressive increase from the control level up to the highest tested concentration of 100 mg/mL. At this concentration, with CHT_5_, POD activity reached 5.710 ± 0.230 nM/min/g FW, nearly doubling from the control’s activity of 3.410 ± 0.070 nM/min/g FW. This substantial increase underscores a robust enhancement in the oxidative stress response of *P. graveolens*, and suggests that higher concentrations of CHT are particularly effective in activating the defensive mechanisms, indicating its potential as a powerful elicitor in stress response studies.

In contrast, SA-induced changes in POD activity showed a different pattern, peaking notably at a concentration of 25 µM (SA_2_). Following this peak, the activity slightly declined and then plateaued at higher concentrations, indicating a potential threshold beyond which no further enhancement in enzyme activity is observed. The maximal activity induced by SA_2_ was higher compared to the peak activity achieved with CHT_5_ treatment with a POD activity of 5.98 ± 0.03 nM/min/g FW, suggesting that SA might be a more potent elicitor of POD in these cultures, at lower concentrations.

For SOD activity, CHT treatment demonstrated a progressive increase with increasing concentrations, culminating at 100 mg/mL, indicating an optimal elicitor concentration that maximizes the oxidative stress defense with an activity of 0.810 ± 0.012 nM/min/mg FW, superior of that of the non-treated calli from the control group with only 0.550 ± 0.009 nM/min/mg FW. Conversely, the response of SOD activity to SA-elicited callus cultures peaked at the second concentration of 25 µM with an activity way superior than the control group with an activity of 0.982 ± 0.024 nM/min/mg FW.

These findings highlight the distinctive influences of CHT and SA on the oxidative enzymatic defenses in *P. graveolens* calli cultures, which are crucial for understanding the stress response pathways activated by these elicitors. The results not only underscore the potential application of such elicitors in enhancing the production of secondary metabolites through induced stress but also provide a comparative insight into the efficacy and potential mechanisms of action of these two commonly used plant defense elicitors.

Previous studies have indicated that chitosan and salicylic acid can significantly enhance the activities of SOD and POD in cell suspension and callus cultures across various plant species (Zaragoza-Martínez et al., 2016; Woch et al., 2023; Fan et al., 2010; Khan et al., 2021). A report by Fan et al. (2010), indicates the involvement of POD and SOD in mediating chitosan-induced stress in cell suspension cultures of *Betula platyphylla* Suk (Fan et al., 2010).

Khan et al. (2019), reported that SOD and POD activities were elevated prior to and further enhanced by elicitation with salicylic acid and chitosan in callus cultures of *Fagonia indica* Burm. f. Similarly, Woch et al. (2023), observed that elicitation with salicylic acid and jasmonic acid significantly boosted the antioxidant enzymatic defenses, specifically SOD, catalase (CAT), and POD, as well as non-enzymatic defense mechanisms including ascorbate, total glutathione, and reduced glutathione (GSH).

The results of our study underscore the specific roles of CHT, and SA in regulating reactive oxygen species as systemic signaling molecules, enabling plants to mitigate biotic and abiotic stress. Among the treatments, CHT_5_ and SA_2_ demonstrated the highest levels of activity, which were positively associated with increased biomass as well as elevated polyphenol and flavonoid content observed in earlier findings (Du Jardin, 2015; Zheng and Zhu, 2003; Wasternack and Parthier, 1997).

### 3.4 Antioxidant activity

Plants maintain their equilibrium by neutralizing free radicals with phytochemicals that function as antioxidants (Li et al., 2016; Girgih et al., 2024; Engwa, 2018). Phenolic compounds are one of the primary classes of these molecules known for scavenging free radicals (Sánchez‐Moreno et al., 1998).

**FIGURE 6:**
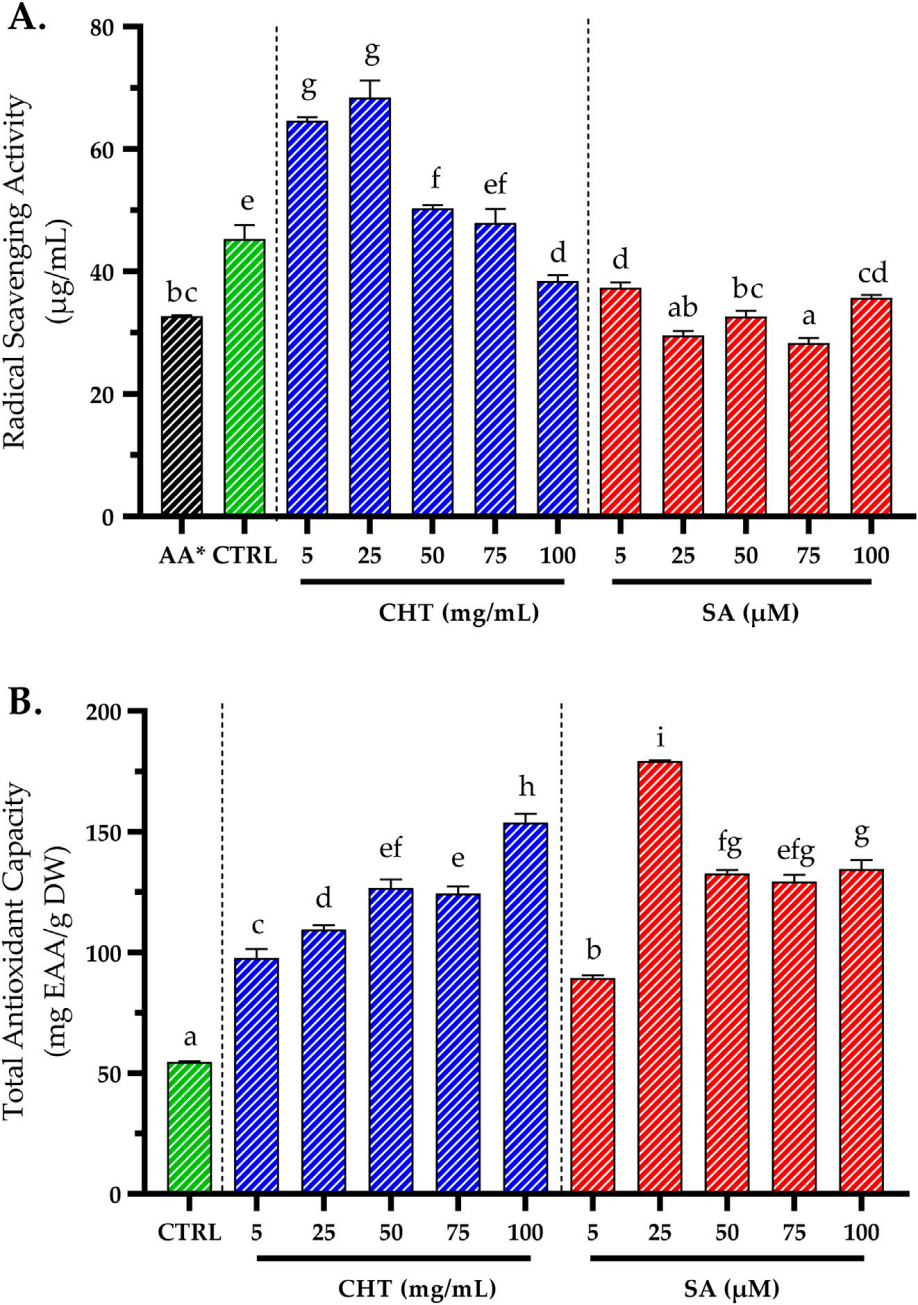
The impact of varying concentrations of chitosan (CHT) and salicylic acid (SA) on the antioxidant activity of *P. graveolens* callus cultures using DPPH assay **(A)**, and total antioxidant capacity **(B)**. Results are expressed as means ± standard error, with different letters denoting statistically significant differences as determined by the Tukey test (*p* ≤ 0.05). * The values were compared to the DPPH standard (IC_50_ of ascorbic acid), which was measured at 32.73 ± 0.09 μg/mL.

Antioxidant activity is commonly evaluated using the DPPH assay, which measures the ability of a sample to scavenge free radicals, with the results expressed as the half-inhibitory concentration (IC_50_) (Elbouzidi et al., 2023). In this study, the lowest IC_50_ values, indicating higher antioxidant activity, were observed in SA_2_-and SA_3_-treated cultures, with values of 29.56 ± 0.74 μg/mL and 32.63 ± 0.94 μg/mL, respectively. These were followed by CHT_5_-treated cultures, which showed an IC_50_ of 38.44 ± 0.96 μg/mL, while the control group exhibited a comparatively higher IC_50_ of 45.31 ± 2.31 μg/mL (Figure 6A). The superior antioxidant activity observed in SA- and CHT-treated cultures is primarily attributed to the elevated levels of phenolics and flavonoids, which are well-established contributors to the antioxidant capacity of plants (Akbari et al., 2022; Hano and Tungmunnithum, 2020).

The Total Antioxidant Capacity (TAC) was also evaluated using the phosphomolybdenum method, which measures the reduction of Mo(VI) to Mo(V) by the antioxidants present in the sample. This reduction leads to the formation of a green phosphate/Mo(V) complex under acidic conditions. This assay is particularly effective for detecting various antioxidants, including phenolics, ascorbic acid, α-tocopherol, and carotenoids (Prieto et al., 1999). The results revealed significantly higher TAC values in CHT_5_-and SA_2_-treated cultures, measuring 153.85 ± 3.54 mg EAA/g DW and 179.22 ± 0.32 mg EAA/g DW, respectively, compared to the control, which showed a much lower value of 54.60 ± 0.32 mg EAA/g DW (Figure 6B).

The enhanced antioxidant activities in SA- and CHT-treated cultures are likely due to the stimulation of genes encoding antioxidant enzymes, such as superoxide dismutases (SODs), catalases, and ascorbate peroxidases, which protect cells from reactive oxygen species (ROS)-induced damage (Lucho-Constantino et al., 2017; Ali et al., 2006; Chong et al., 2005). Previous studies have also reported increased antioxidant activity in plants treated with CHT or SA, further supporting these findings (Chong et al., 2005; Piotrowska et al., 2010).

### 3.5 Tyrosinase inhibition

Tyrosinases, identified as EC 1.14.18.1, belong to the type 3 copper protein family and are alternatively known as polyphenol oxidases (Pillaiyar et al., 2017). These enzymes catalyze two sequential reactions in the presence of molecular oxygen: firstly, the hydroxylation of phenols into *o*-diphenols, known as monophenolase activity, and secondly, the oxidation of these *o*-diphenols to *o*-quinones, referred to as diphenolase activity. The *o*-quinones produced are highly reactive and spontaneously polymerize into melanin, contributing to skin pigmentation and the browning of fruits and vegetables (Momtaz et al., 2008). Consequently, inhibiting tyrosinase activity is a pivotal strategy in reducing melanogenesis, widely utilized in depigmentation treatments. Given the common skin aging symptoms such as wrinkles, roughness, and various types of hyperpigmentation including age spots and melasma, there is a growing demand for skin-lightening cosmetics. With the aim of circumventing the skin irritation often associated with synthetic ingredients, the exploration of natural alternatives has become increasingly important.

**FIGURE 7:**
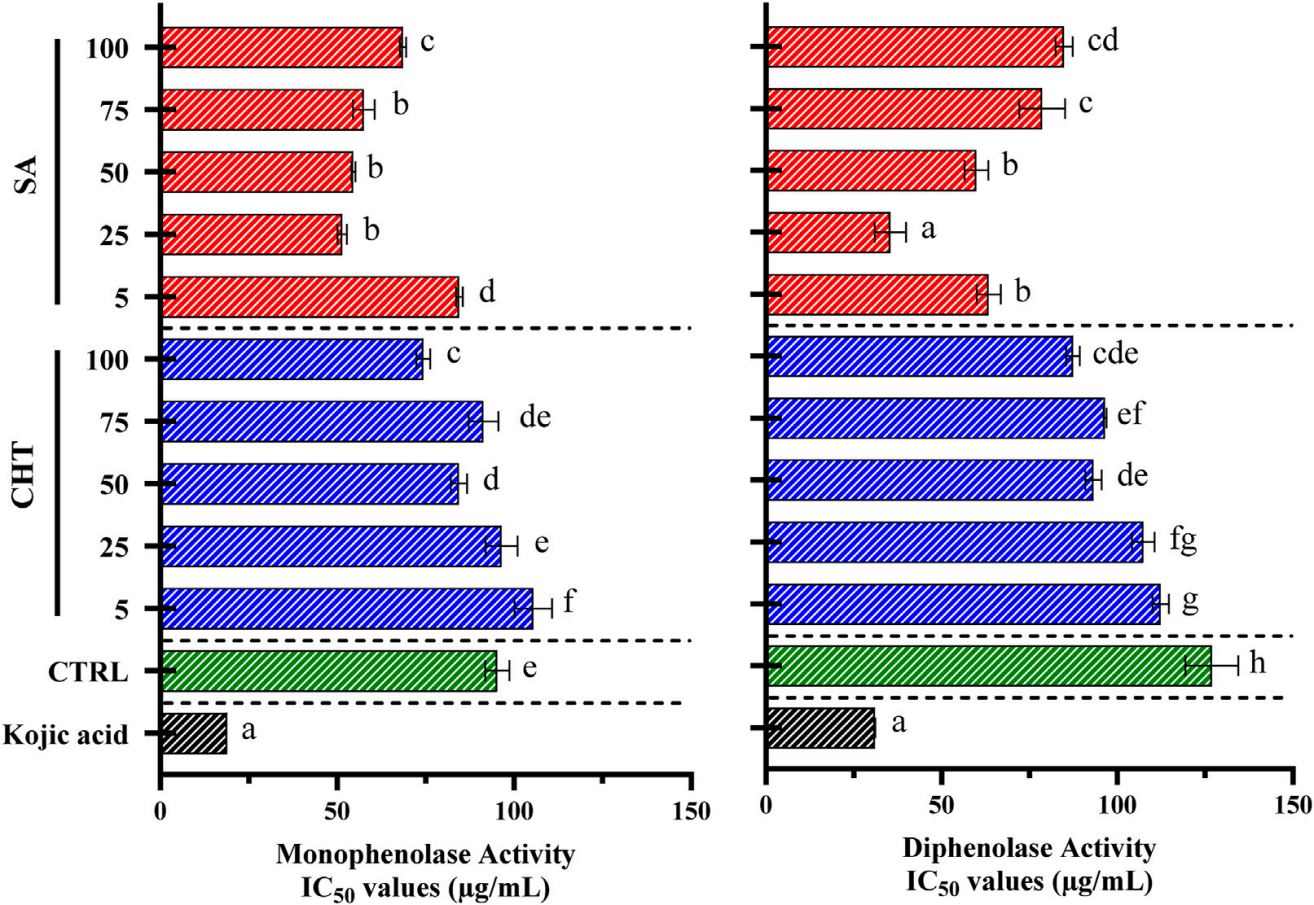
The activities of monophenolase and diphenolase in callus extracts were evaluated using mushroom tyrosinase. L-DOPA was used as the substrate to measure monophenolase activity, whereas L-tyrosine was employed to assess diphenolase activity. Data are shown as the mean ± standard deviation, derived from three separate experiments. Statistical significance is denoted by different letters when *p*-values are less than 0.05.

In our research, we investigated the impact of callus extracts on the monophenolase and diphenolase activities of mushroom tyrosinase, focusing on extracts derived from calli treated with SA, and CHT, alongside a control group without elicitation (Figure 7). The goal was to determine how elicitation influences the phenolic composition of the calli and their capacity to regulate tyrosinase activity, which is vital for controlling melanin synthesis pathways and has implications for both pharmaceutical and cosmetic applications. Our findings revealed that extracts from calli elicited with chitosan (specifically CHT_3_ and CHT_5_) and salicylic acid (SA_2_) showed a significant increase in the inhibition of tyrosinase activities compared to the control. Notably, monophenolase activity was substantially inhibited by extracts from CHT_5_ and SA_2_, displaying IC_50_ values of 74.31 ± 1.93 μg/mL and 51.43 ± 1.31 μg/mL, respectively. In comparison, kojic acid, a well-established tyrosinase inhibitor, demonstrated the most potent inhibition with an IC_50_ value of 18.92 ± 0.02 μg/mL. In the case of diphenolase activity, while CHT_3_ and CHT_5_ also exhibited strong inhibitory effects, SA_2_ presented the most effective inhibition with an IC_50_ value of 35.42 ± 4.42 μg/mL, significantly lower than the control’s 74.31 ± 1.93 μg/mL. Kojic acid again showed the highest inhibition with an IC_50_ value of 31.04 ± 0.09 μg/mL.

These findings indicate that elicitation with CHT_5_, and SA_2_ induces a favorable response in *P. graveolens* callus cultures, resulting in significant tyrosinase inhibition. This effect can be attributed to the enhanced biosynthesis of phenolic compounds influenced by these elicitors. The study highlights those extracts derived from *P. graveolens* calli treated with CHT or SA possess potent tyrosinase inhibitory properties, demonstrating their potential for application in skin-whitening formulations in the cosmetic industry.

### 3.6 Elastase inhibition

**FIGURE 8:**
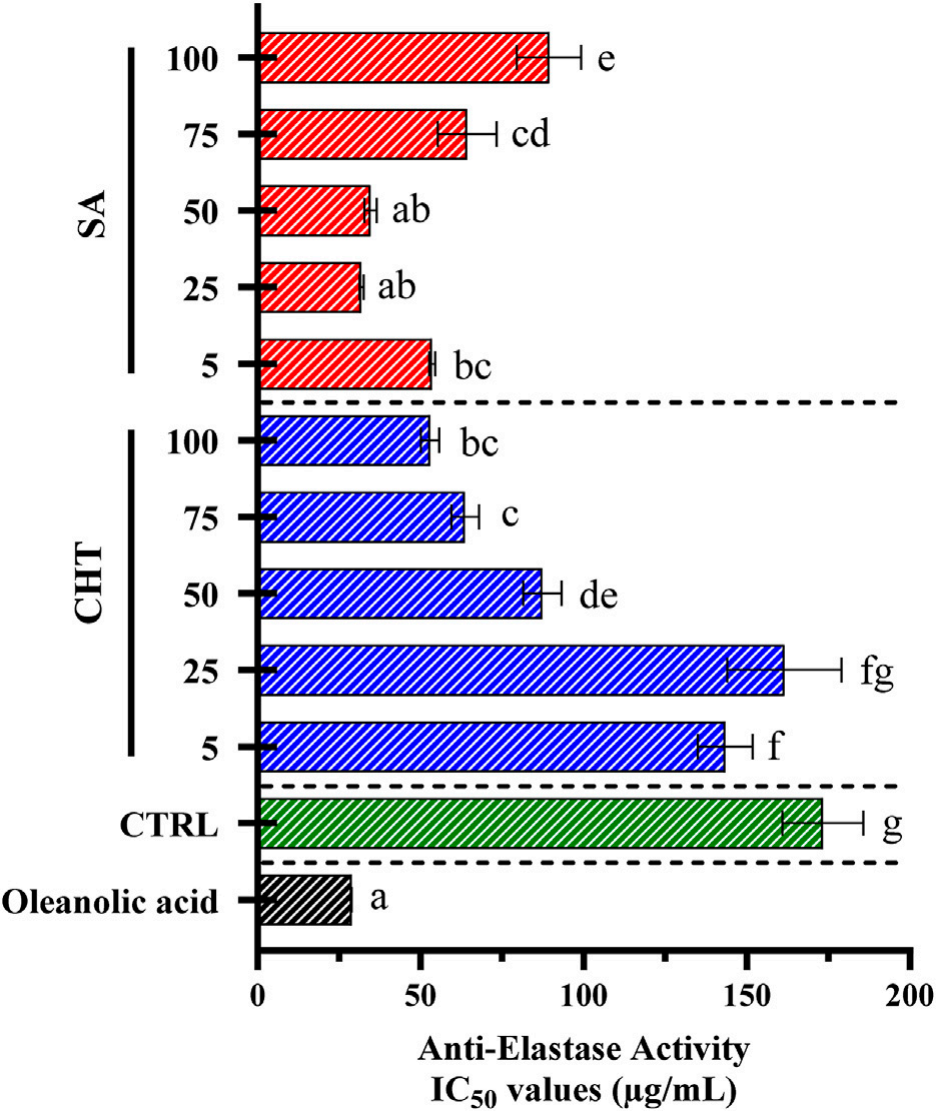
The anti-elastase efficacy of callus extracts was determined using porcine pancreatic elastase. The results are expressed as mean ± standard deviation, based on data from three independent experiments. Statistically significant variations are marked by different letters at a significance level of *p* < 0.05.

Elastase is a proteolytic enzyme involved in breaking down the skin’s elastic fibers, a process that accelerates skin aging and reduces elasticity (Panwar et al., 2020). The inhibition of elastase is crucial to preserving elastin fibers, maintaining skin firmness, and slowing the onset of visible aging signs (Imokawa and Ishida, 2015). In this study, elastase activity was assessed using the substrate N-Succ-Ala-Ala-Ala-p-nitroanilide, as shown in Figure 8.

Figure 8 presents the anti-elastase activity of callus extracts treated with SA, and CHT at various concentrations, as well as a control group (CTRL) and oleanolic acid as a reference standard. The anti-elastase activity was measured in terms of IC_50_ values. Lower IC_50_ values indicate stronger inhibitory activity.

The anti-elastase activity of callus extracts treated with SA and CHT demonstrates a clear concentration-dependent response. Cultures treated with the highest concentration of CHT_5_ exhibit a strong inhibitory effect, with an IC_50_ value of 52.89 ± 2.78 μg/mL, significantly lower than other concentrations of CHT. This result suggests that higher concentrations of CHT effectively enhance the anti-elastase activity of the extracts. Suggesting that CHT enhances the production of bioactive compounds in callus cultures, contributing to moderate anti-elastase effects (Thring et al., 2009; Kacem, 2013). However, at lower concentrations of SA (25 μg/mL and 50 μg/mL), the inhibitory potential of elastase was slightly higher, ranging from 31.84 ± 0.60 to 34.67 ± 1.93 μg/mL significant inhibition compared to CHT-treated group and the non-treated group (IC_50_ = 173.31 ± 12.42 μg/mL). These findings are consistent with previous studies showing that SA can upregulate the production of phenolic and flavonoid compounds, which are known to inhibit elastase activity by blocking proteolytic enzymes (Radjah et al., 2021; Jakimiuk et al., 2021). Oleanolic acid, used as a reference standard, shows a highly potent inhibitory effect with an IC_50_ value of 28.82 ± 0.02 μg/mL. This strong activity underscores oleanolic acid’s established role as an elastase inhibitor and serves as a benchmark for assessing the efficacy of the callus extracts in inhibiting elastase (Nema et al., 2013).

The results demonstrate that both salicylic acid and chitosan effectively enhance the anti-elastase activity of callus extracts, with salicylic acid showing a superior effect, particularly at lower concentrations. This suggests that elicitation with these agents can significantly modulate the production of bioactive compounds capable of inhibiting elastase, an enzyme implicated in skin aging and inflammatory conditions. The anti-elastase activity is likely due to the increased synthesis of phenolic compounds and flavonoids, which have been reported to possess strong elastase inhibitory effects by binding to the active site of the enzyme and disrupting its activity (Mainka et al., 2021).

The findings are consistent with the broader literature on plant elicitation, where treatments such as salicylic acid and chitosan are employed to boost the production of secondary metabolites, thereby enhancing the bioactivity of plant extracts. These results underscore the potential of using elicitation strategies in plant tissue culture to develop extracts with targeted therapeutic properties, particularly for applications in skincare and anti-aging formulations (Bouzroud et al., 2023).

### 3.7 Sun protection factor against ultra-violet radiation

UV radiation is universally recognized as the predominant cause of extrinsic skin aging, often termed photoaging. This process involves the accelerated deterioration of the skin resulting from extended and repeated exposure to the sun’s UV rays (Baumann, 2007; Mbanga et al., 2014). Clinically, it is characterized by symptoms such as the appearance of wrinkles, reduced skin elasticity, uneven pigmentation, and the formation of various skin lesions (Taylor, 2005). On a molecular level, these changes are driven by the breakdown of collagen, increased activity of matrix metalloproteinases, and elevated oxidative stress, which collectively compromise the structural integrity and function of the skin.

Chronic exposure to ultraviolet radiation (UVR) is also strongly associated with a range of skin disorders, including the development of various types of skin cancer, such as basal cell carcinoma, squamous cell carcinoma, and melanoma (Lohani et al., 2019). Ultraviolet radiation (UVR) is divided into three types according to their wavelengths: UVA (320–400 nm), UVB (290–320 nm), and UVC (100–290 nm). UVC radiation is largely blocked by the Earth’s atmosphere, but UVA and UVB rays can penetrate the skin, playing significant roles in photoaging and the development of skin cancer (Rastogi et al., 2010). UVB rays are particularly harmful as they directly damage DNA, potentially leading to mutations that may result in skin cancer. On the other hand, UVA rays reach deeper into the dermis where they generate reactive oxygen species, leading to oxidative stress that can further damage the skin and accelerate aging (Rastogi et al., 2010). Together, UVA and UVB synergistically accelerate both visible and molecular signs of skin aging, underlining the need for protective strategies to mitigate their harmful effects.

In assessing the UVB-blocking efficacy of various treatments, our study meticulously measured the absorbance across the UVB spectrum ranging from 290 nm to 320 nm for control (CTRL), chitosan-elicited calli (CHT_5_), and salicylic acid-elicited calli (SA_2_) (Table 4). The absorbance data collected was utilized to compute the Sun Protection Factor (SPF) value, employing the formula developed by Mansur et al. (1986b). The CTRL samples, representing non-elicited calli, provided a baseline for minimal UV protection, which was significantly enhanced in samples treated with chitosan and salicylic acid. The spectrophotometric data revealed a clear gradation in performance. Chitosan (CHT) treatments exhibited moderate absorbance values, suggesting the compound’s intrinsic UV protective properties, possibly due to its film-forming capabilities which scatter UV light (Korać and Khambholja, 2011). Salicylic acid (SA) treatments demonstrated superior absorbance across all wavelengths tested, confirming its robust UV absorption characteristics (Mansur et al., 1986a). These findings are consistent with similar studies highlighting the effectiveness of phenolic acids, and flavonoids in sunscreen formulations due to its capacity to absorb high-energy UVB rays effectively (Cefali et al., 2019; Monsalve-Bustamante et al., 2020).

**TABLE 4:** Absorbance data of hydroalcoholic aliquots of CHT_5_, SA_2_, and CTRL, and their calculated SPF, and %UVB blocked.

Wavelength (nm)	EE(λ) x I(λ) ** Employed	Absorbance *		
		CTRL	CHT_5_	SA_2_
290	0.0150	0.6473 ± 0.0274	0.7127 ± 0.0212	0.9310 ± 0.0083
295	0.0817	0.5977 ± 0.0069	0.6627 ± 0.0497	0.7493 ± 0.0246
300	0.2874	0.7717 ± 0.0227	0.8207 ± 0.0090	0.9083 ± 0.0119
305	0.3278	0.7040 ± 0.0219	0.9707 ± 0.0281	1.0707 ± 0.1089
310	0.1864	0.8103 ± 0.0204	0.8103 ± 0.0204	1.7517 ± 0.0889
315	0.0837	0.3670 ± 0.0110	0.6430 ± 0.0064	0.9883 ± 0.1253
320	0.0180	0.6083 ± 0.0714	0.7290 ± 0.0229	0.7983 ± 0.0141
Calculated SPF		7.038 ± 0.207^a^	8.369 ± 0.208^b^	11.108 ± 0.686^c^
% UVB Blocked ***		85.79%	88.05%	90.99%

*Values are shown as the mean absorbance ±standard deviation based on three readings (n = 3); ** these constant values reflect the erythemogenic effect (EE) of radiation at wavelength λ combined with the solar intensity (I) at that wavelength, as established by (Sayre et al., 1979). *** The percentage of UVB blocked was computed using the method described in Section 2.8. Distinct letters signify statistically significant differences as identified by One-way ANOVA followed by Tukey’s *post hoc* test at p < 0.05.

The calculated SPF values corresponded directly with the absorbance data, where CTRL showed the lowest SPF of 7.038 ± 0.207, indicative of its minimal protective properties. CHT_5_ treated samples displayed an SPF of 8.369 ± 0.208, while SA_2_ treated samples reached an SPF of 11.108 ± 0.686, the highest among the treatments. This finding supports the potential integration of natural extracts into dermatological formulations to enhance UV protection significantly.

Furthermore, the percentage of UVB radiation blocked was 85.79% for CTRL, 88.05% for CHT_5_, and 90.99% for SA_2_. These percentages underscore the potential of both CHT and SA as effective UVB blockers, with SA showing the highest efficacy. The higher blocking percentage of SA aligns with its increased absorbance and SPF values, suggesting its superior capability to protect against UVB radiation, which is critical for preventing skin damage and reducing the risk of skin cancer (Mansur et al., 1986a; Garcia-Molina et al., 2007).

Given these promising results, future research should explore the synergistic effects of combining these extracts in sun protection formulations. Such studies could lead to the development of sunscreens with optimized SPF values and enhanced UV protection, potentially offering broader protection against the deleterious effects of sun exposure. Additionally, examining other natural or synthetic compounds that might synergize with CHT and SA-elicited calli could further advance the field of photoprotective skincare products.

### 3.8 Correlation analysis

**FIGURE 9:**
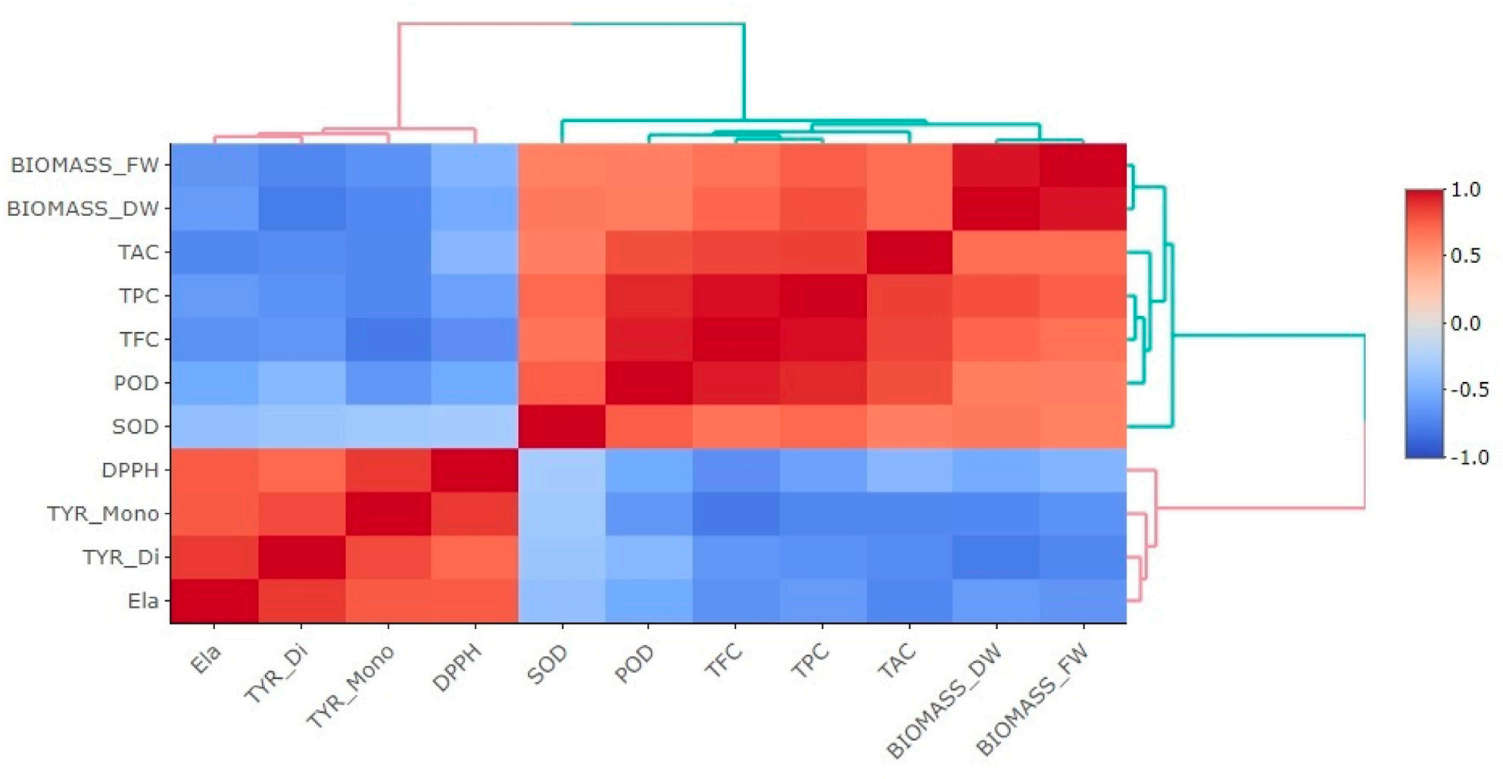
The correlogram illustrates the relationships between various examined parameters and their biological activities. Correlations with p-values exceeding 0.05 are considered not significant. The diagram uses colored rectangles to represent the correlation coefficients: negative correlations are shown in various shades of blue, with darker blue representing the strongest negative correlation (where r = −1), and positive correlations are displayed in shades of red, from light to dark red, with dark red denoting the weakest positive correlation (where r = 1). Parameters included are DPPH, representing 2,2-diphenyl-1-picrylhydrazyl radical scavenging activity (IC_50_ values considered), TYR_Mono and TYR_Di for monophenolase and diphenolase activities of tyrosinase inhibition (IC_50_ values considered), Ela for elastase inhibition (IC_50_ values considered), TPC for total polyphenols content, TFC for total flavonoid content, POD for peroxidase activity, SOD for superoxide dismutase activity, TAC for total antioxidant content, and BIOMASS_DW and BIOMASS_FW for changes in dry and fresh biomass, respectively.

The correlogram presented in Figure 9 depicts a Pearson correlation analysis that was used to examine the relationships among the levels of secondary metabolites, activities of antioxidant enzymes, and the biological effects observed in elicited *P. graveolens* cultures. This analysis includes parameters such as total antioxidant capacity (TAC), superoxide dismutase (SOD) activity, peroxidase (POD) activity, total flavonoid content (TFC), total phenolic content (TPC), fresh and dried biomass (Biomass_FW and Biomass_DW, respectively), tyrosinase inhibitory activities for monophenolase (TYR_Mono) and diphenolase (TYR_Di), anti-elastase activity (Ela), and DPPH radical scavenging activity (DPPH). The color gradients in the correlogram represent the strength and direction of the correlations, with blue shades indicating negative correlations and red shades indicating positive correlations. Darker colors correspond to stronger correlations, whether positive or negative.

The correlogram reveals several significant positive correlations among the variables. Notably, TPC and TFC exhibit strong positive correlations (r = 0.85, r = 0.84; at *p* < 0.01, and *p* < 0.001, respectively) with TAC, as indicated by the deep red shades. This suggests that higher phenolic and flavonoid contents are closely associated with enhanced antioxidant capacity. These findings are consistent with existing literature, which emphasizes the significant role of phenolic and flavonoid compounds in contributing to the antioxidant properties of plant extracts through their free radical scavenging abilities and redox properties (Kahramanoğlu et al., 2019; Rice-Evans et al., 1997). Similarly, positive correlations between biomass (both fresh and dry) and TPC, TFC, and TAC suggest that increased biomass accumulation is linked to higher production of polyphenols and flavonoids, thereby enhancing the overall antioxidant capacity of the cultures. This trend indicates that elicitor treatments that promote biomass growth can also enhance secondary metabolite synthesis, supporting their dual role in plant defense and growth regulation (Verma and Shukla, 2015).

Conversely, several negative correlations are observed, particularly between SOD activity and DPPH radical scavenging activity, as highlighted by the deep blue hues. This inverse relationship suggests that higher SOD activity is associated with reduced DPPH scavenging efficiency, possibly due to a shift in antioxidant mechanisms from non-enzymatic (free radical scavenging) to enzymatic pathways in response to oxidative stress (Apel and Hirt, 2004). This observation aligns with the idea that plants may adaptively modulate their antioxidant strategies depending on the type and intensity of stress encountered, balancing between enzymatic and non-enzymatic antioxidants (Mittler, 2002). Additionally, negative correlations between elastase, and tyrosinase inhibitory activities (Ela, TYR_Di and TYR_Mono), and TPC and TFC suggest that higher phenolic and flavonoid contents is directly correspond to enhanced enzyme inhibition (which corresponds to a lower IC_50_ values). This could reflect the complexity of interactions between different bioactive compounds and specific enzyme inhibition pathways, where other structural factors of phenolics, rather than their overall abundance, might play a critical role in tyrosinase inhibition (Kim and Uyama, 2005).

The correlogram also highlights distinct clustering patterns among the variables, indicating closely related functional groups. For example, TPC, TFC, and TAC are grouped together, emphasizing their interconnected roles in enhancing antioxidant activities. In contrast, SOD and POD cluster separately, reflecting their synergistic involvement in enzymatic antioxidant defense mechanisms. These clusters provide valuable information about the coordinated responses of secondary metabolites and enzymes under elicitation, revealing how specific treatments can modulate multiple biochemical pathways simultaneously.

Overall, this analysis underscores the complex and dynamic relationships between secondary metabolites, antioxidant enzymes, and biological activities in elicited cultures of *P. graveolens*. The strong positive correlations between phenolic and flavonoid contents with antioxidant capacity highlight the importance of these compounds in enhancing the bioactivity of plant extracts, consistent with numerous studies that have demonstrated the health-promoting effects of these phytochemicals (Shahidi and Naczk, 2003; Balasundram et al., 2006). The observed negative correlations between different antioxidant mechanisms suggest a strategic balance in plant stress responses, wherein enzymatic and non-enzymatic antioxidants complement each other to maintain cellular redox homeostasis. These findings support the use of elicitation as a biotechnological approach to optimize both growth and bioactive compound production in medicinal plants, enhancing their therapeutic potential (Ramirez-Estrada et al., 2016).

## 4 Conclusion, limitations, and study perspectives

The present study demonstrates the significant impact of chitosan and salicylic acid as elicitors in optimizing the production of bioactive compounds in *P. graveolens* callus cultures. Both elicitors effectively promoted the accumulation of phenolic and flavonoid compounds, with SA_2_ at 25 µM resulting in the highest TPC (242.09 ± 0.18 µg GAE/g DW) and CHT_5_ at 100 mg/mL showing substantial TFC enhancement (156.53 ± 0.41 µg GAE/g DW). Enhanced antioxidant activities were observed, as indicated by significant reductions in IC_50_ values for DPPH radical scavenging and improved total antioxidant capacity. Notably, SA and CHT treatments also significantly boosted anti-tyrosinase and anti-elastase activities. These results underscore the effectiveness of SA and CHT in stimulating secondary metabolite synthesis through upregulation of enzymatic and non-enzymatic defense pathways, making them valuable tools in plant tissue culture for cosmeceutically relevant compound production.

Future research should focus on optimizing elicitor concentrations and exposure durations to maximize the yield of bioactive compounds. Exploring the combined effects of multiple elicitors could also enhance the overall efficacy of elicitation strategies. Additionally, expanding this approach to other plant species and targeting different classes of secondary metabolites would broaden the potential applications of elicitation in biotechnology. Integrating advanced omics technologies, such as metabolomics and transcriptomics, will be essential in uncovering the molecular mechanisms behind elicitor-induced responses, providing deeper insights into how these treatments modulate plant metabolism. These findings could pave the way for innovative and sustainable production methods of high-value phytochemicals for applications in pharmaceuticals, cosmetics, and functional foods, ultimately contributing to the advancement of natural product development.

## Data Availability

The original contributions presented in the study are included in the article/Supplementary Material, further inquiries can be directed to the corresponding authors.
