# Supplementary material for: Elicitor-driven enhancement of phenolic compounds in geranium callus cultures: phytochemical profiling via LC-MS/MS and biological activities
Source: Front Chem. 2025 Mar 7;13:1537877. doi: 10.3389/fchem.2025.1537877 (PMC11925866; doi:10.3389/fchem.2025.1537877)
Supplement: Supplementary file 1 [file DataSheet1.docx]

**Supplementary file**

**Elicitor-Driven Enhancement of Phenolic Compounds in Geranium Callus Cultures: Phytochemical Profiling via LC-MS/MS and Biological Activities**


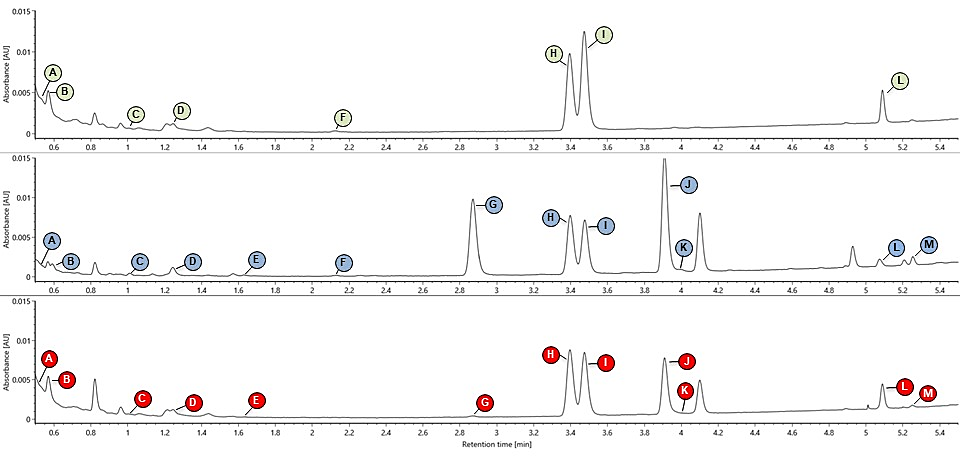


TIC Control - *P. graveolens*

TIC Salicylic acid (25µM) - *P. graveolens*

TIC Chitosan (100 mg/mL) - *P. graveolens*

**Panel A**

.

**Figure S1.** TIC chromatograms at 280 nm display the control sample (green labels), the SA-elicited cultures (blue labels), and the CHT-elicited cultures (red labels). Panel A covers retention times from 0.5 to 5.5 minutes. The compounds identified include: **(A)** Gallic acid, **(B)** 3-Hydroxybenzoic acid, **(C)** 4-Hydroxybenzoic acid, **(D)** Caffeic acid, **(E)** Vanillic acid, **(F)** Syringic acid, **(G)** 3-Hydroxycinnamic acid, **(H)** Salicylic acid, **(I)** Ferulic acid, **(J)** Rutin, **(K)** Sinapic acid, **(L)** *p*-Coumaric acid, **(M)** Cinnamic acid.


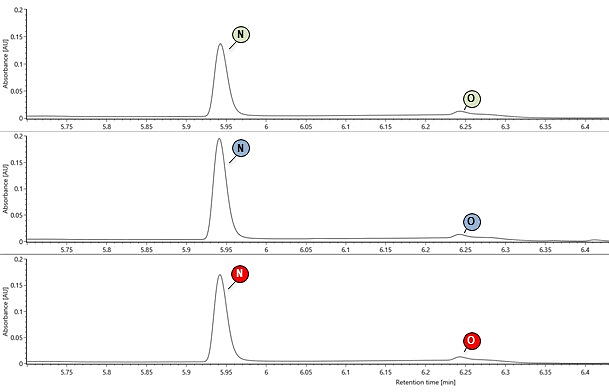


TIC Control - *P. graveolens*

TIC Salicylic acid (25µM) - *P. graveolens*

TIC Chitosan (100 mg/mL) - *P. graveolens*

**Panel B**

**Figure S2.** TIC chromatograms at 280 nm display the control sample (green labels), the SA-elicited cultures (blue labels), and the CHT-elicited cultures (red labels). Panel B spans 5.5 to 6.45 minutes. The compounds identified include: **(N)** Kaempferol, and **(O)** *trans*-Chalcone.

**Table S1.** UV quantification of the most abundant phenolic compounds in the elicited cell cultures.

| **Labels** | **Compounds** | **Quantity (mg/ 100 g DW of sample)** | | |
| --- | --- | --- | --- | --- |
|  |  | **CTRL** | **SA (25 µM)** | **CHT (100 mg/mL)** |
| **A** | Gallic acid | 1.3591 ± 0.0085 | 1.6758 ± 0.0040 | 1.3783 ± 0.0250 |
| **B** | 3-hydroxybenzoic acid | 97.4853 ± 10.1739 | 25.9973 ± 6.0549 | 99.8320 ± 0.0000 |
| **C** | 4-Hydroxybenzoic acid | 13.8760 ± 3.9361 | 12.1173 ± 2.2622 | 9.0000 ± 7.6728 |
| **D** | Caffeic acid | 7.0620 ± 0.0028 | 6.0420 ± 0.0028 | 7.3427 ± 0.1672 |
| **E** | Vanillic acid | - | 0.2894 ± 0.0035 | 0.1354 ± 0.0012 |
| **F** | Syringic acid | 0.2613 ± 0.0514 | 0.1213 ± 0.0211 | - |
| **G** | 3-hydroxycinnamic acid | - | 38.7090 ± 2.2046 | 0.9064 ± 0.1824 |
| **H** | Salicylic acid | 21.0680 ± 1.8472 | 13.4740 ± 0.3026 | 20.3987 ± 2.8386 |
| **I** | Ferulic acid | 27.8460 ± 1.3721 | 22.8356 ± 0.3729 | 25.3757 ± 0.9733 |
| **J** | Rutin | - | 30.6360 ± 3.0023 | 21.1040 ± 2.2340 |
| **K** | Sinapic acid | - | 0.6820 ± 0.1141 | 0.2998 ± 0.0292 |
| **L** | *p*-Coumaric acid | 0.3014 ± 0.0000 | 0.2623 ± 0.0000 | 0.2411 ± 0.0000 |
| **M** | Cinnamic acid | - | 0.7567 ± 0.0220 | 0.1880 ± 0.0212 |
| **N** | Kaempferol | 103.6787 ± 5.0099 | 192.8160 ± 17.9977 | 119.6773 ± 12.0129 |
| **O** | *trans*-chalcone | 43.4817 ± 6.8965 | 41.3814 ± 3.4268 | 40.7632 ± 0.2081 |
| **Total Phenolics (mg/100 g DW)** | | **316.42 ± 14.03** | **387.79 ± 19.79** | **346.64 ± 14.74** |

Data are means ± standard error; -: trace, below LOQ.

**Table S2.** Validation characteristics data of the phenolic standards, and sensitivity of the UV quantification approach.

| **Labels** | **Phenolic compounds** | **Retention time (min)** | **R² ^a^** | **Linear range (mg/mL)** | **LOD^b^ (mg/mL)** | **LOQ^c^ (mg/mL)** |
| --- | --- | --- | --- | --- | --- | --- |
| **A** | Gallic acid | 0.56 | 0.99 | 0.001–1 | 0.02 | 0.05 |
| **B** | 3-hydroxybenzoic acid | 0.68 | 0.99 | 0.001–1 | 0.05 | 0.16 |
| **C** | 4-Hydroxybenzoic acid | 1.04 | 0.99 | 0.001–1 | 0.01 | 0.03 |
| **D** | Caffeic acid | 1.28 | 0.99 | 0.001–1 | 0.03 | 0.09 |
| **E** | Vanillic acid | 1.63 | 0.99 | 0.001–1 | 0.01 | 0.02 |
| **F** | Syringic acid | 2.21 | 0.99 | 0.001–1 | 0.01 | 0.03 |
| **G** | 3-hydroxycinnamic acid | 2.92 | 0.99 | 0.001–1 | 0.01 | 0.05 |
| **H** | Salicylic acid | 3.43 | 0.99 | 0.001–1 | 0.02 | 0.05 |
| **I** | Ferulic acid | 3.51 | 0.99 | 0.001–1 | 0.03 | 0.09 |
| **J** | Rutin | 3.93 | 0.99 | 0.001–1 | 0.02 | 0.06 |
| **K** | Sinapic acid | 4.00 | 0.99 | 0.001–1 | 0.03 | 0.08 |
| **L** | *p*-Coumaric acid | 5.14 | 0.99 | 0.001–1 | 0.01 | 0.04 |
| **M** | Cinnamic acid | 5.31 | 0.99 | 0.001–1 | 0.02 | 0.05 |
| **N** | Kaempferol | 5.94 | 0.99 | 0.001–1 | 0.05 | 0.08 |
| **O** | trans-chalcone | 6.26 | 0.99 | 0.001–1 | 0.03 | 0.09 |

a, Correlation coefficients of the regression equation. b, LOD limit of detection. c, LOQ limit of quantification.
